# Panx1 and P2X7R are associated with impaired skeletal health and delayed bone development in early onset of type 1 diabetes

**DOI:** 10.3389/fendo.2026.1829463

**Published:** 2026-05-08

**Authors:** Zeynep Seref-Ferlengez, Marcia Urban-Maldonado, Aylin Uyar, Zar C. Thent, Angela Tun, Bridget P. Saw, Mitchell B. Schaffler, Sylvia O. Suadicani, Mia M. Thi

**Affiliations:** 1 Departments of Orthopedic Surgery, Albert Einstein College of Medicine and Montefiore Medical Center, Bronx, NY, United States; 2 Departments of Urology, Albert Einstein College of Medicine and Montefiore Medical Center, Bronx, NY, United States; 3 Departments of Molecular Pharmacology, Albert Einstein College of Medicine and Montefiore Medical Center, Bronx, NY, United States; 4 Department of Biomedical Engineering, City College of New York, New York, NY, United States

**Keywords:** bone development, early-life endocrinology, inflammation, NLRP3 inflammasome, osteocyte mechanosignaling, Panx1-P2X7R complex, skeletal fragility, type 1 diabetes

## Abstract

**Objectives:**

Type 1 diabetes (T1D) is a chronic autoimmune endocrine disorder that disrupts multiple physiological systems beyond glucose homeostasis. An underrecognized complication is impaired skeletal development during childhood and adolescence, a critical period for bone accrual. This study aimed to define the early-life effects of T1D on load-induced bone adaptation and to investigate the role of the osteocyte Panx1-P2X7R mechanosignaling complex in diabetes-associated skeletal deficits.

**Materials and methods:**

Akita (C57BL/6J-Ins2^Akita^) and age-matched wild-type mice were subjected to treadmill loading (1, 2, or 4 weeks; 5 days/week, 300 m/day) or maintained under normal cage activity. Femoral bone density and anabolic responses were assessed using *in vivo* imaging and dynamic histomorphometry. In parallel, osteocytes cultured under normal or high-glucose (HG) conditions were exposed to oscillatory fluid shear stress (OFSS; 1, 3, or 5 days) to model mechanical loading *in vitro*. Temporal changes in Panx1-P2X7R signaling components and inflammatory mediators in bone tissue and cultured osteocytes were analyzed by Western blot and quantitative PCR.

**Results:**

Load-induced periosteal bone formation in wild-type mice was accompanied by adaptive regulation of Panx1-P2X7R expression, whereas this response was disrupted in young adult T1D mice. Dysregulation emerged after one week of loading and was recapitulated in osteocytes exposed to OFSS *in vitro* under high glucose conditions. Diabetic bone and HG-conditioned osteocytes exhibited sustained inflammatory activation, including upregulation of the NLRP3 inflammasome and proinflammatory cytokines (IL-1β, TNF-α). The failure of Panx1-P2X7R to adapt to mechanical loading, together with persistent inflammation, suggests a mechanistic link between impaired mechanotransduction and inflammasome activation in T1D.

**Conclusion:**

Early-onset T1D impairs bone mechanoadaptation and is associated with dysregulated Panx1-P2X7R signaling and heightened inflammatory responses. Given the established roles of Panx1 and P2X7R in mechanosensitive signaling and inflammation, their disruption likely contributes to defective skeletal adaptation and inflammatory bone pathology, ultimately increasing skeletal fragility in T1D.

## 1 Introduction

Bone loss in people with type 1 diabetes (T1D) is a diabetic complication that is often overlooked. Studies have shown that T1D children and adults exhibit low bone mineral density (BMD), along with a reduction in bone mass ranging from 5% to over 21% (1–4). Given that T1D typically manifests early in adolescence, insufficient accrual of peak bone mass and impaired bone formation are likely to significantly contribute to compromising the skeletal integrity of individuals with T1D. Childhood and adolescence represent crucial phases for skeletal development, making individuals with early-onset T1D particularly susceptible to skeletal alterations (5, 6). As T1D patients age, their susceptibility to osteoporosis and bone lesions significantly increases, leading to a higher incidence of osteoporotic fractures (3–5, 7). Despite growing clinical and epidemiological evidence supporting the association of T1D with low bone mass and fractures, the mechanisms underlying diabetic osteopenia are still not completely understood.

The effects of T1D on bone mechanical and material properties have been widely investigated in animal models. These studies have revealed that in rodent models of T1D, the decline observed in bone mechanical properties is similar to what has been observed in diabetic people: weaker bones with lower fracture energy, lower bending, and torsional strength (8–11). In adult T1D animal models, multiple alterations in the bone matrix have been reported, including reduced mineral crystal size, collagen glycation, diminished canalicular wall and osteocyte process areas, and increased osteocyte apoptosis. These changes, together with elevated osteocalcin levels, may collectively contribute to impaired bone matrix quality and the associated increases in skeletal fragility and fracture risk observed in diabetes (11–16). Moreover, adult T1D mice, either Akita (spontaneous T1D model) or streptozotocin (STZ, chemically induced), exhibit an impaired anabolic response to mechanical loading (17, 18). A proper response to mechanical loading is essential for maintaining bone health, and the inability of bone cells to respond to mechanical challenges imposed on the bone may play a central role in the mechanisms underlying skeletal dysfunction in T1D.

Osteocytes are the most abundant and the primary mechanosensitive cells in bone. They sense and integrate both mechanical and chemical signals from their lacunar-canalicular environment, initiating and coordinating the responses of effector cells (osteoblasts and osteoclasts) required for the local regulation of both bone formation and resorption (19, 20). Osteocytes are significantly affected by exposure to high glucose (HG) levels, one of the hallmarks of T1D (18, 21–24). In addition to increasing the cell size and microfilament density, HG has been shown to increase expression of sclerostin, an anti-anabolic mediator, and induce apoptosis, while decreasing expression of the connexin 43 (Cx43) gap junction, a mechanosignaling mediator, and reducing flow-induced PGE_2_ release from osteocytes (17, 22–24). Osteocytes’ exposure to HG also reduces flow-induced ATP release, a key bone mechanosignaling molecule, through mechanisms involving the mechanosensitive pannexin 1 channel (Panx1) and purinergic P2X7 receptor (P2X7R).

Panx1 and P2X7R are components of the osteocyte mechanosome and form a functional complex that provides a major pathway for mechanosensitive ATP release (21). Our previous studies demonstrated that Panx1-P2X7R expression is significantly reduced in bones from T1D Akita mice and following *in vitro* exposure of osteocytes to HG, leading to impaired Panx1-P2X7R-mediated flow-induced ATP release from these cells (21). The critical roles of P2X7R and Panx1 in bone homeostasis are further supported by studies using transgenic mouse models. The bone anabolic response to mechanical loading is reduced in both P2X7R- and Panx1-deficient mice (25, 26). Collectively, these findings suggest that the osteocyte Panx1-P2X7R complex is a key mediator in mechanisms driving bone dysfunction in diabetes (21, 27).

In addition to its role in mechanosignaling, the Panx1-P2X7R complex has also been implicated in inflammatory responses across various cell types. Panx1 has been shown to participate in innate immune responses by interacting with P2X7R and components of the NLRP3 inflammasome (28). The NOD-like receptor pyrin domain containing 3 (NLRP3), together with the adaptor protein apoptosis-associated speck-like protein containing a CARD (PYCARD or ASC), recruits pro-caspase-1 to form the NLRP3 inflammasome, resulting in caspase-1 activation and subsequent processing of pro-IL-1β and pro-IL-18 into their mature pro-inflammatory cytokine forms, IL-1β and IL-18 (29–31). Likewise, P2X7R has also been shown to mediate IL-1β maturation mainly through the recruitment of the NLRP3 inflammasome associated with processes such as lysosome secretion, exosome release, and pyroptosis (32–34). Diabetes is often associated with chronic low-grade inflammation, and inflammatory conditions have been recognized as contributing factors to bone loss (35, 36). Shortly after the onset of diabetes in STZ-induced T1D mice, local inflammatory cytokine expression in bone is elevated and subsequently declines toward normal levels as diabetes progresses, concurrently with changes in bone phenotypic markers (Runx2, osteocalcin) (37, 38). Dysregulation of the Panx1-P2X7R could be one of the factors driving the local inflammatory responses observed in the T1D bone.

In this study, we examined the effects of early-onset diabetes on skeletal growth and explored the role of the osteocyte Panx1-P2X7R mechanosignaling complex in mechanisms mediating load-induced bone adaptation and altered local inflammatory responses during a critical period of skeletal development. Using the Akita mouse model, we found that diabetes compromised cortical bone integrity and anabolic responses essential for normal skeletal growth, which were associated with dysregulated load-induced Panx1-P2X7R signaling and elevated local inflammation. These alterations were recapitulated *in vitro* when osteocytes were exposed to diabetes-like conditions. Collectively, our findings identify the Panx1-P2X7R complex as a key mediator of the detrimental effects of early-onset diabetes on the developing skeleton, linking impaired anabolic adaptation to both NLRP3 inflammasome activation and proinflammatory cytokine–driven inflammation.

## 2 Materials and methods

### 2.1 Animals

Young adult type 1 diabetic Akita mice (C57BL/6-Ins2^Akita^/J) and wild-type (Wt) littermates at 5 to 12 weeks of age were used in this study to capture the critical time window of skeletal growth that coincides with the early onset of diabetes. Akita heterozygote mice were purchased from the Jackson Laboratory (Bar Harbor, ME; strain # 003548) and bred in our animal facility. The diabetic phenotype in Akita male mice is more severe, progressive, and uniform than in females, and not all Akita females become diabetic. Therefore, in this study, we only used males to consistently present signs of hyperglycemia at early stages of skeletal development. Male Wt offspring from Akita breeding were used as controls. All mice were allowed free access to standard mouse chow (5058-PicoLab Mouse Diet 2; LabDiet, CA) and water and were housed on a light/dark (12h/12h) cycle at 23 °C during the whole experimental period.

### 2.2 *In vivo* bone density measurements

Femur bone density of Akita and Wt mice was measured at 5, 8, 12, 16, 20, 24, and 28 weeks of age using the *In vivo* Imaging System FX Pro (Carestream, Rochester, NY). At each time point, mice were anesthetized by intraperitoneal injection of a ketamine (KETATHESIA; Henry Schein, Melville, NY) and xylazine (AnaSed, Akorn Inc, Lake Forest, IL) mixture (150:10 mg/kg) and X-ray images were acquired with the following settings: Aluminum filter: 0.8 mm, camera setting: *f2.8*, exposure time: 10 sec. Bone density was measured from the femoral midshaft (both femurs/mouse) using Carestream Bone Density Software Module as previously described (39–41). Body weight measurements were recorded at each time point.

### 2.3 Mechanical loading paradigm

Littermate Akita and Wt mice were exposed to a treadmill loading paradigm as we previously described (26). We chose treadmill running to impose mechanical loading because of its physiological relevance. Briefly, 8-week-old mice were randomly divided into two groups: mechanical loading (loaded) and cage control (non-loaded). The mechanical loading group was subjected to treadmill running for 1 or 2, or 4 weeks (5 days/week, total of 300 meters/day, speed 10 meter/min using Columbus Instruments, Model 1055M, Columbus, OH) as previously described (26, 42). All mice were allowed normal cage activity between loading sessions.

### 2.4 Tissue and blood collection

At the experimental endpoint, mice were placed in an induction chamber (1 L) and exposed to 5% isoflurane (Pivetal, Loveland, CO) for 2–3 min, until loss of reflexes was observed. Terminal blood samples were collected via cardiac puncture using a 25–27 G needle inserted into the left ventricle. Blood was aspirated slowly, transferred to appropriate tubes for serum preparation, and animals were subsequently euthanized by cervical dislocation. Blood glucose was measured using a Contour Next One glucometer (Ascensia Diabetes Care, Parsippany, NJ). Hindlimbs were collected from 8-week-old cage control mice to serve as a baseline reference group (0 week, prior to treadmill running). Experimental samples were then obtained from both treadmill-running and cage control groups following the final running bout at 1, 2, or 4-week timepoints. The hindlimbs were processed for molecular analyses. Briefly, the femora and tibiae were cleaned of soft tissue. To isolate osteocyte-enriched samples, the epiphyses were removed, and the remaining diaphyses were centrifuged to remove the bone marrow, as previously described (21, 43). The diaphyses were then homogenized using Mixer Mill MM 400 (Retsch USA, Newton, PA) for protein or RNA extraction, followed by qPCR and Western blotting analysis.

### 2.5 Western blotting

Osteocyte-enriched homogenized bone samples were sonicated in lysis buffer (5 mM EDTA, 1 mM Na Orthovanadate, 1 mM NaHCO_3_, 2 mM PMSF [Sigma-Aldrich], and 1× protease inhibitor [Roche, Mannheim, Germany]) and electrophoresed on 10% SDS-PAGE gels for protein separation and transferred to nitrocellulose membranes (Whatman GmbH, Dassel, Germany). The membranes were probed with primary polyclonal antibodies to P2X7R (1:1,000; APR-004, Alomone Labs, Israel), Panx1 (N [Term], 1:100; Cat 487900, Invitrogen Corporation, CA), and β-actin (1:20,000; A1978, Sigma-Aldrich) followed by incubation with respective horseradish peroxidase (HP)-conjugated anti-rabbit IgG and anti-mouse IgG (1:10,000; Santa Cruz Biotechnology, TX). The protein bands were detected using the Immobilon Western detection kit (Millipore, MA) as previously described (21). Densitometric analyses were performed using ImageJ (NIH) software. Measured intensities for all samples were first normalized with respective loading controls, β-actin, and with either age-matched non-loaded Wt or age-matched non-loaded Akita or age-matched loaded Wt bone tissue samples.

### 2.6 Reverse transcription quantitative PCR

qPCR was used to quantify mRNA expression levels of the Panx1 and P2X7R mechanosignaling complex, NLRP3 inflammasome components (NLRP3, ASC, and Casp1), and inflammatory mediators IL-1β and TNF-α in non-loaded and loaded Wt and Akita bones. Total RNA was extracted using TRIzol reagent (Invitrogen) in combination with Rneasy Plus Kit (Qiagen). qPCR was performed using SYBR GREEN Master Mix (Applied Biosystems, CA) in an Applied Biosystems 7300 Real-Time PCR System (Forester City, CA), according to the manufacturer’s instructions and as previously described (44). Briefly, 0.5 μg of total RNA was reverse transcribed into cDNA using SuperScript VILO (Invitrogen). RT reactions were diluted in RNase-free water to a 1:50 ratio, and 3 µl of this dilution was used in amplification reactions carried out for 40 cycles with an annealing temperature of 60°C in a 25μl final volume. Finally, a dissociation profile of the PCR product(s) was obtained by a temperature gradient running from 60°C to 95°C. Forward and reverse primer sequences are listed in **Supplementary Table 1** (45–48). We performed two technical replicas for each sample, with a total of six to seven biological replicas per experimental group. Relative gene expression levels were calculated using the delta-delta CT method, where values obtained for the gene of interest are first normalized to those of the reference gene, 18S ribosomal RNA, and subsequently to the average of their respective age-matched controls.

### 2.7 Histomorphometric analysis

Dual intraperitoneal injections of calcein (30 mg/kg body weight, Sigma-Aldrich, St. Louis, MO) were administered to the treadmill running and cage control groups at 3 and 10 days after the first loading day for the 2-week timepoint group and at 3 and 16 days after the first loading day for the 4-week timepoint group. Animals were euthanized immediately after the last running bout at 2, or 4-week timepoints, the femurs were harvested, fixed in 10% neutral buffered formalin (Fisher Scientific, PA), stained en-bloc with Villanueva staining, dehydrated in graded ethanol, and embedded undecalcified in poly-methyl methacrylate (PMMA), as previously described (49). The mid-diaphysis was then cross-sectioned using a diamond wafering saw (Leica SP1600 microtome, Leica Biosystems, IL) and polished to 90 μm, and two sections per femur were examined using OsteoMeasure (OsteoMetrics, GA) in a Zeiss microscope (Carl Zeiss, CA) with a 10X objective. Total perimeter (B.Pm), single label perimeter (sL.Pm), double label perimeter (dL.Pm), double label area (dL.Ar), and inter labeled width (Ir.L.Wi [μm]) were measured from periosteal (Ps) and endocortical (Ec) surfaces. Cortical bone area [Ct.B.Ar (mm^2^)], total area [Tt.Ar (mm^2^)], marrow area [Ma.Ar (mm^2^)], cortical thickness [Ct.Th (μm)], polar moment of inertia (*J* [mm^4^]) and area moment inertia (I_x_ and I_y_ [mm^4^]) were also measured. Following parameters were calculated as previously described using ImageJ (NIH) (50, 51): mineralizing surface {MS/BS (%): [(sL.Pm+dL.Pm)/2B.Pm]×100%}; mineral apposition rate [MAR (μm/day): Ir.L.Wi/7]; bone formation rate [BFR/BS (μm^3^/μm^2^/day): MARxMS/BS] Measurements were made by a single observer (ZS‐F) who was blinded to specimen identification.

### 2.8 Assessment of osteocyte integrity in mid-shaft femur

Osteocyte apoptosis was assessed as described previously (49, 52). Briefly, normal osteocytes (fully occupying lacunae, N.Ocy/B.Ar, #/mm^2^) and atypical appearing osteocytes (pyknotic or retracted, A.Ocy/B.Ar, #/mm^2^) were quantified in basic fuchsin-stained, undecalcified ground mid-shaft femur sections from non-loaded and loaded Wt and Akita mice. Brightfield images were acquired at 64X magnification. For each sample, measurements were obtained from three randomly selected regions, with three to five images acquired per region.

### 2.9 Cell culture and fluid flow experiments

MLO-Y4 osteocytes (53) were cultured in μ-slide I^0.4^ chamber (ibidi GmbH, Germany) in α-MEM supplemented with 1% penicillin-streptomycin, 5% fetal bovine serum, and 2.5% bovine serum (Invitrogen Corporation, Carlsbad, CA). Cultures were maintained in a humidified incubator at 37 °C with 5% CO_2_. Cells were seeded at 10^3^ cells/cm^2^, and 24 hours later, they were serum starved for 12 hours to induce cell cycle synchronization (54). Cells were then cultured for 10 days under three distinct conditions: in control normal glucose [NG: Medium with 100 mg/dL (5.5 mM) glucose], in high glucose [HG: Medium with 450 mg/dL (25 mM) glucose] and in mannitol (Man: Medium with 100 mg/dL glucose + 350 mg/dL mannitol) as previously described (21). Mannitol, a nonabsorbable sugar, was used as a control for differences in osmolarity and possible associated effects. Media were changed every other day. Oscillatory fluid shear stress (OFSS, τ = ± 10 dyne/cm^2^) at 1 Hz was applied to the cells under sterile condition for 1, 3 and 5 days (30-minute bout/day) on a heated plate (37°C) using Legato 200 syringe pump (KD Scientific, MA, USA) and a syringe filled with NG or HG or Man supplemented media. Samples were collected immediately after the OFSS exposure for qPCR analysis.

### 2.10 Statistical analysis

Data were analyzed using ImageJ (NIH) and Prism 9 software (GraphPad, CA). Factor correction method was used to eliminate between-session or between-run variation in replicated experiments for Western blot and qPCR (55, 56). Statistical differences in longitudinal bone density and body weight between wild-type and Akita mice were determined using two-way ANOVA followed by Sidak’s multiple comparisons test. Statistical differences between different loaded and non-loaded samples were determined by one-way ANOVA followed by Tukey’s or Dunnett’s multiple comparison test. For comparisons between two unpaired groups, data were first tested for normality using the Shapiro-Wilk test. If the data met the assumptions of normality, Welch’s t-test was used; otherwise, the nonparametric Mann–Whitney U test was applied. P < 0.05 was considered statistically significant.

## 3 Results

### 3.1 Effect of type 1 diabetes on juvenile bone density

**Figure 1:**
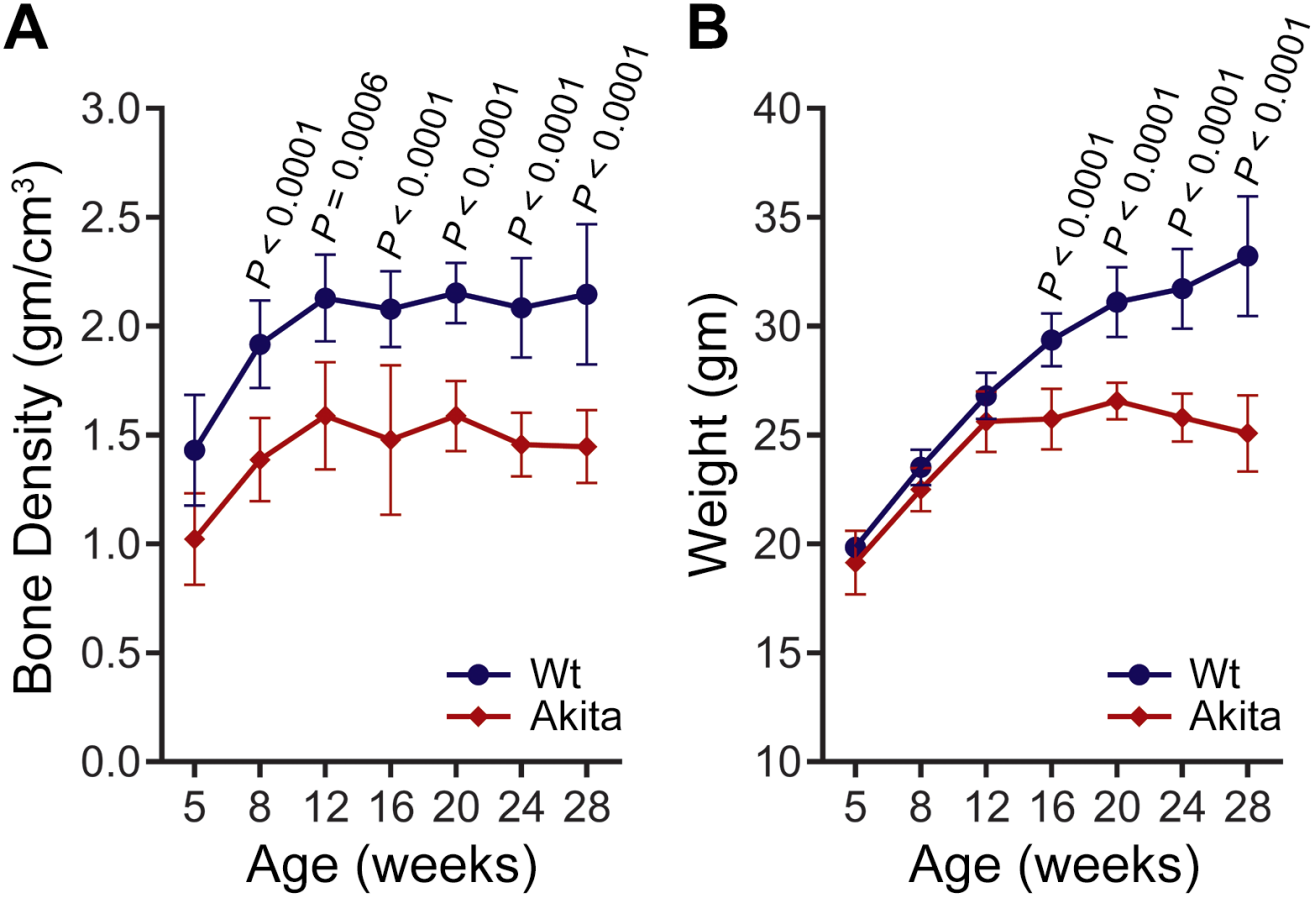
Bone density of T1D Akita mice is significantly lower than that of their age-matched wild-type during skeletal development and throughout adulthood. Longitudinal comparison of **(A)** femur (midshaft) bone density and **(B)** body weight of wild-type (Wt) and T1D Akita mice. *P* values for Akita vs. Wt at each time point were obtained by two- way ANOVA followed by Sidak’s multiple comparisons test. n = 4 (5 weeks); n = 9 (8, 12, and 16 weeks), n = 7–9 (20, 24, and 28 weeks). Data presented as means ± SD.

To investigate the impact of T1D onset on bone during skeletal development, we performed a temporal assessment of bone density in Wt and T1D Akita mice starting at 5 weeks and ending at 28 weeks of age. As shown in **Figure 1A**, bone density was significantly affected by diabetes, starting as early as 8 weeks of age in T1D Akita mice, which corresponds to 4–5 weeks after the onset of the disease. Compared with age-matched Wt controls, T1D Akita mice exhibited bone density that was 15% lower at 8 weeks of age, and up to 28% lower by 28 weeks of age. Notably, after peaking at 12 weeks, bone density plateaued at a significantly lower level rather than continuing to decline. Although the body weight was not significantly affected by diabetes during skeletal development (5 to 12 weeks of age), the body weight of Akita mice was markedly lower than those of their age-matched Wt once the skeleton reached maturity (**Figure 1B**). This finding shows that manifestation of diabetes at early adolescence leads to early and significant complications on skeletal development and health that persist throughout adulthood.

### 3.2 Load-induced bone response is impaired in young adult T1D Akita mice

Lower bone density detected in Akita mice at early developmental stages was accompanied by, and potentially attributable to, an impaired anabolic response. Previous studies have shown that adult T1D Akita mice (28-week-old) display reduced anabolic response to mechanical loading (18). We have now examined the time course of the development of this impaired response, and whether it coincides with the onset of juvenile diabetes and the critical time of skeletal growth. We found that during skeletal development (8–12 weeks of age), although young adult T1D Akita mice do not show overt skeletal abnormalities, they nevertheless exhibit significantly lower bone density and altered bone parameters compared with age-matched WT mice. During this time frame, there were clear signs of diabetes in the Akita mice as indicated by significantly higher non-fasting blood glucose levels when compared to age-matched Wt (**Table 1**). At 12 weeks of age, the body weight and Ma.Ar of cage control, Akita and Wt mice were not different. However, some femoral midshaft parameters were significantly smaller in cage control Akita mice [e.g., (Ct.B.Ar (-12%), Ct.B.Ar/Tt.Ar (-11%), and Ct.Th (-13%)]. Following 4 weeks of treadmill running, we observed that Wt mice responded to mechanical loading with a marked increase in Tt.Ar (16%), Ct.B.Ar (12%), Ma.Ar (21%), Ct.Th (4%), *J* (41%)*, Ix* (36%), and *Iy* (43%) compared to non-loaded Wt, indicating an anabolic response (radial expansion). In contrast, this load-induced anabolic response was mostly absent in T1D Akita mice when compared to their non-loaded Akita counterparts. Moreover, in Akita mice, the load-induced changes in Ct.B.Ar (-19%), Ct.B.Ar/Tt.Ar (-14%), and Ct.Th (-15%) were substantially lower when compared to non-loaded Wt mice. Similarly, the robust anabolic responses observed in loaded Wt bones were significantly impaired in loaded Akita bones [Tt.Ar (-19%), Ct.B.Ar (-27%), Ct.B.Ar/Tt.Ar (-9%), Ct.Th (-19%), *J* (- 42%)*, Ix* (-39%), and *Iy* (-43%)]. No apparent T1D-induced or load-induced osteocyte apoptosis was observed in the young adult Akita femur (**Supplementary Figure 1**).

**Table 1:** Animal characteristics and histological parameters showing load-induced changes in the femoral midshaft of 12-week-old young adult Wild-type and T1D Akita bones after 4 weeks of loading.

Parameters	Wildtype non-loaded (n=7)	Wildtype loaded (n=7)	*P*	T1D Akita non-loaded (n=6)	*P*	T1D Akita loaded (n=7)	*P*
**Blood Glucose (mg/dL)**	197 ± 21	204 ± 65	0.99^a^	**550 - >600**	**<0.0001^a,b^**	**550 - >600**	**<0.0001^a,b^**, 0.94^c^
**Body Weight (g)**	26.6 ± 0.9	25.8 ± 2.12	0.85^a^	23.9 ± 2.97	0.09^a^, 0.34^b^	**22.9 ± 1.57**	**0.01^a^**, **0.06^b^**, 0.81^c^
**Tt.Ar (mm^2^)**	1.95 ± 0.06	**2.26 ± 0.21**	**0.0051^a^**	**1.91 ± 0.19**	0.97^a^, **0.0032^b^**	**1.83 ± 0.19**	0.57^a^, **0.0002^b^**, 0.87^c^
**Ct.B.Ar (mm^2^)**	0.86 ± 0.06	**0.97 ± 0.06**	**0.021^a^**	**0.75 ± 0.09**	**0.043^a^**	**0.70 ± 0.07**	**0.0009^a^**, **<0.0001^b^**, 0.53^c^
**Ct.B.Ar/Tt.Ar**	0.45 ± 0.03	0.42 ± 0.03	0.31^a^	**0.39 ± 0.02**	**0.035^a^**, 0.54^b^	**0.38 ± 0.04**	**0.0049^a^**, 0.17^b^, 0.91^c^
**Ma.Ar (mm^2^)**	1.08 ± 0.07	**1.31 ± 0.19**	**0.016^a^**	1.15 ± 0.12	0.81^a^, 0.19^b^	1.13 ± 0.15	0.91^a^, 0.092^b^, 0.99^c^
**Ct.Th (μm)**	196.5 ± 13	204.5 ± 14	0.67^a^	**170.3 ± 10**	**0.0078^a^**, **0.005^b^**	**166.6 ± 13**	**0.0016^a^**, **<0.0001^b^**, 0.96^c^
***J* (mm^4^)**	0.44 ± 0.05	**0.62 ± 0.09**	**0.0009^a^**	**0.39 ± 0.08**	0.63^a^, **<0.0001^b^**	**0.36 ± 0.07**	0.21^a^, **<0.0001^b^**, 0.88^c^
***I_x_* (mm^4^)**	0.14 ± 0.02	**0.19 ± 0.05**	**0.0275^a^**	**0.13 ± 0.03**	0.90^a^, **0.0074^b^**	**0.12 ± 0.02**	0.50^a^, **0.001^b^**, 0.91^c^
***I_y_* (mm^4^)**	0.30 ± 0.03	**0.42 ± 0.05**	**0.0002^a^**	**0.26 ± 0.05**	0.48^a^, **<0.0001^b^**	**0.24 ± 0.05**	0.14^a^, **<0.0001^b^**, 0.88^c^

*P* values for comparisons with respect to ^a^non-loaded wildtype, ^b^loaded wildtype and ^c^non-loaded Akita were obtained using one-way ANOVA followed by Tukey’s multiple comparisons test. Bold values indicate parameters that exhibit significant changes. n = number of animals/groups. Total area (Tt.Ar), cortical bone area (Ct.B.Ar), marrow area (Ma.Ar), cortical thickness (Ct.Th), polar moment of inertia (*J*), and area moment of inertia (*I_x_* and *I_y_*) are presented as the means ± SD.

**Figure 2:**
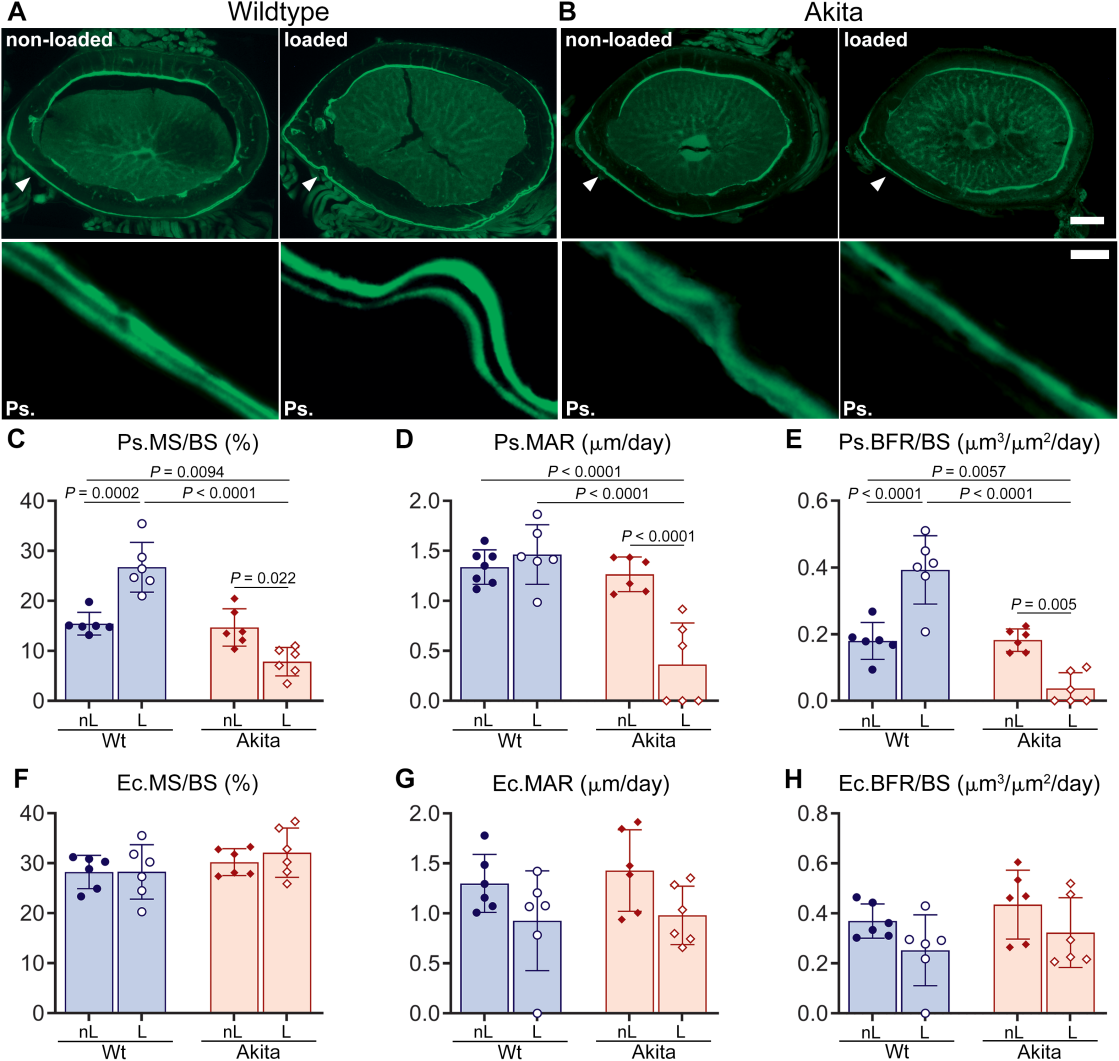
Load-induced changes in the periosteal and endocortical surfaces of the femoral midshaft of young adult wild-type and T1D Akita mice after 2 weeks of loading. Representative cross-sectional images from age-matched non-loaded and loaded femoral midshafts of 10-week-old **(A)** wild-type (Wt) and **(B)** Akita bones, showing double calcein labels (top panel, scale bar = 200 μm). Representative images of double calcein labeling on medial periosteal surfaces (Ps.) corresponding to arrow heads in top panels of non-loaded (nL) and loaded (L) Wt and Akita bones (scale bar = 20 μm). Dynamic histomorphometric studies showing load-induced changes in **(C)** periosteal mineralizing surface (Ps.Ms/Bs), **(D)** periosteal mineral apposition rate (Ps.MAR), **(E)** periosteal bone formation rate (Ps.BFR/BS), **(F)** endocortical mineralizing surface (Ec.Ms/Bs), **(G)** endocortical mineral apposition rate (Ec.MAR), and **(H)** endocortical bone formation rate (Ec.BFR/BS) are presented as means ± SD. *P* values were obtained one-way ANOVA followed by Tukey’s multiple comparison test; n = 6/group.

Differences in load-induced bone formation parameters between Wt and T1D Akita mice were also detected by dynamic histomorphometry. As shown in **Figure 2**, there were no significant differences in periosteal and endocortical surfaces of non-loaded Wt and Akita bones. However, mechanical loading significantly increased periosteal MS and BFR in Wt femurs relative to that of non-loaded Wt (**Figures 2C, E** blue bars: Ps.MS/Bs = 72%, *P* = 0.0002; Ps.BFR/BS = 116%, *P* < 0.0001), while it substantially reduced periosteal MS, MAR and BFR (**Figures 2C–E**, red bars) in Akita femurs relative to that of i) non-loaded Akita (Ps.MS/Bs = -46%, *P* = 0.022; Ps.MAR = -72%, *P* < 0.0001; Ps.BFR/BS = -78%, *P* = 0.005), ii) non-loaded Wt (Ps.MS/Bs = -50%, *P* = 0.0094; Ps.MAR = -73%, *P* < 0.0001; Ps.BFR/BS = -78%, *P* = 0.0057) and iii) loaded Wt (Ps.MS/Bs = -70%, *P* < 0.0001; Ps.MAR = -75%, *P* < 0.0001; Ps.BFR/BS = -90%, *P* < 0.0001). No statistical differences were observed in endocortical MS, MAR and BFR between loaded and non-loaded femurs in both Wt and Akita mice (**Figures 2F–H**).

### 3.3 Diabetes dysregulates the load-induced adaptive response of the bone’s Panx1-P2X7R mechanosignaling complex

**Figure 3:**
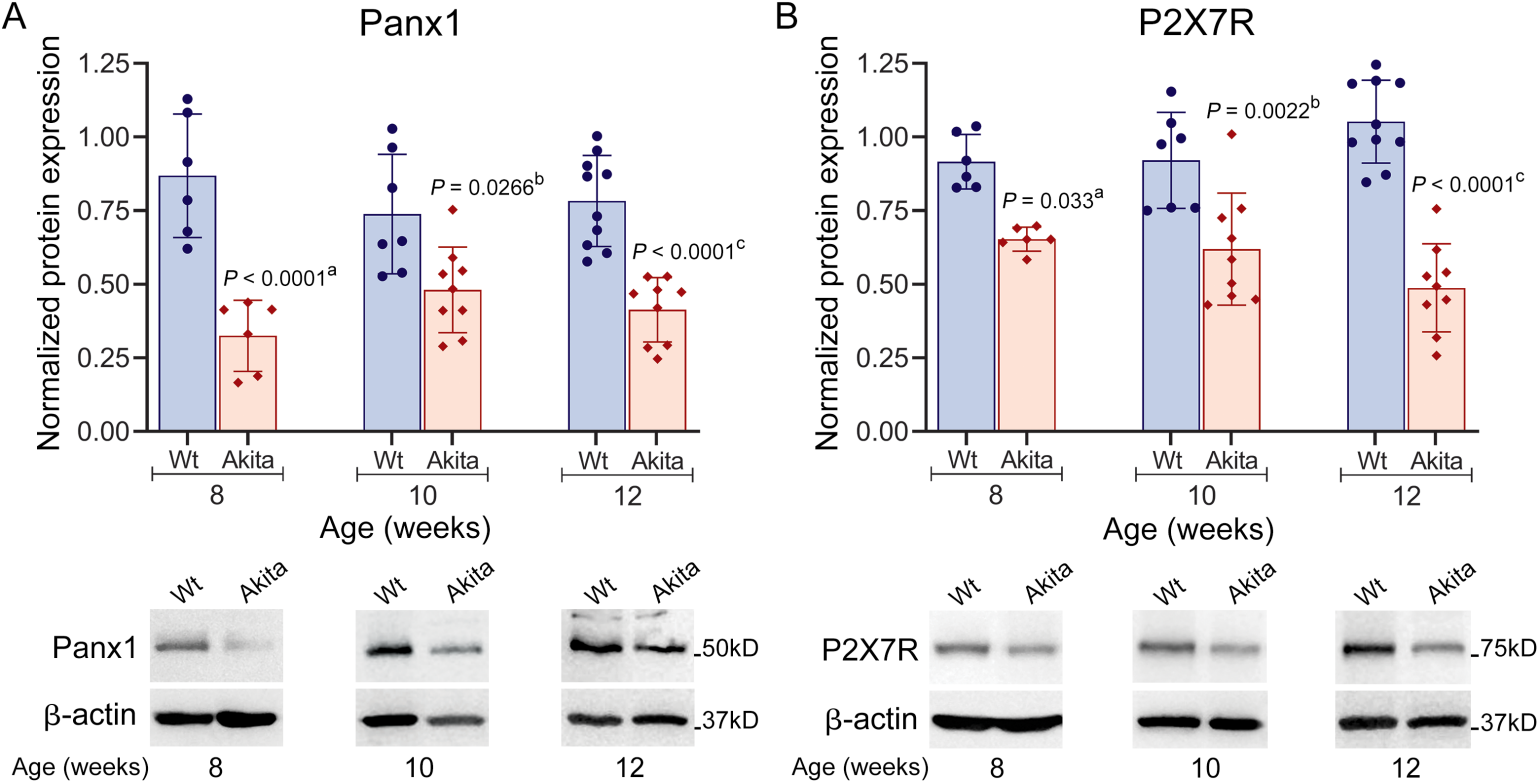
Protein expression levels of Panx1 and P2X7R are significantly lower in young adult T1D Akita mice. Protein expression of **(A)** Panx1 and **(B)** P2X7R in wild-type (Wt) and T1D Akita mice at 8, 10, and 12 weeks of age. Representative Western blots for Panx1 and P2X7R are shown below the graph. Protein levels for all samples were normalized with the respective loading control, β-actin. *P* values for comparisons of Akita at each time point with respect to ^a^8W Wt, ^b^10W Wt, and ^c^12W Wt were obtained using one-way ANOVA followed by Tukey’s multiple comparison test. n = 6 (8 weeks), n= 6–8 (10 weeks), n = 7–9 (12 weeks). Data are presented as the means ± SD.

Our previous studies established that Panx1-P2X7R expression is altered in 8-week-old T1D Akita bones and in osteocytes cultured under T1D-like conditions. Specifically, we demonstrated that HG-induced dysregulation of Panx1-P2X7R complex blunted the osteocyte mechanical responses (21). Given the critical role of ATP mechanosignaling in bone homeostasis, we investigated whether impaired diabetic bone anabolic responses correlate with changes in Panx1-P2X7R protein levels during T1D progression. As shown in **Figure 3**, Panx1 and P2X7R protein expression in Wt bones remains relatively stable during skeletal development. In contrast, the protein levels for both Panx1 and P2X7R were significantly lower in young adult T1D Akita mice. This lower level persisted throughout diabetes progression from 8 to 12 weeks of age when compared to age-matched Wt mice. Given that T1D Akita mice become fully diabetic around the age of 3–4 weeks, our data demonstrates that a short duration of diabetes (4–5 weeks) is sufficient to significantly alter the protein expression of the mechanosignaling Panx1-P2X7R complex.

**Figure 4:**
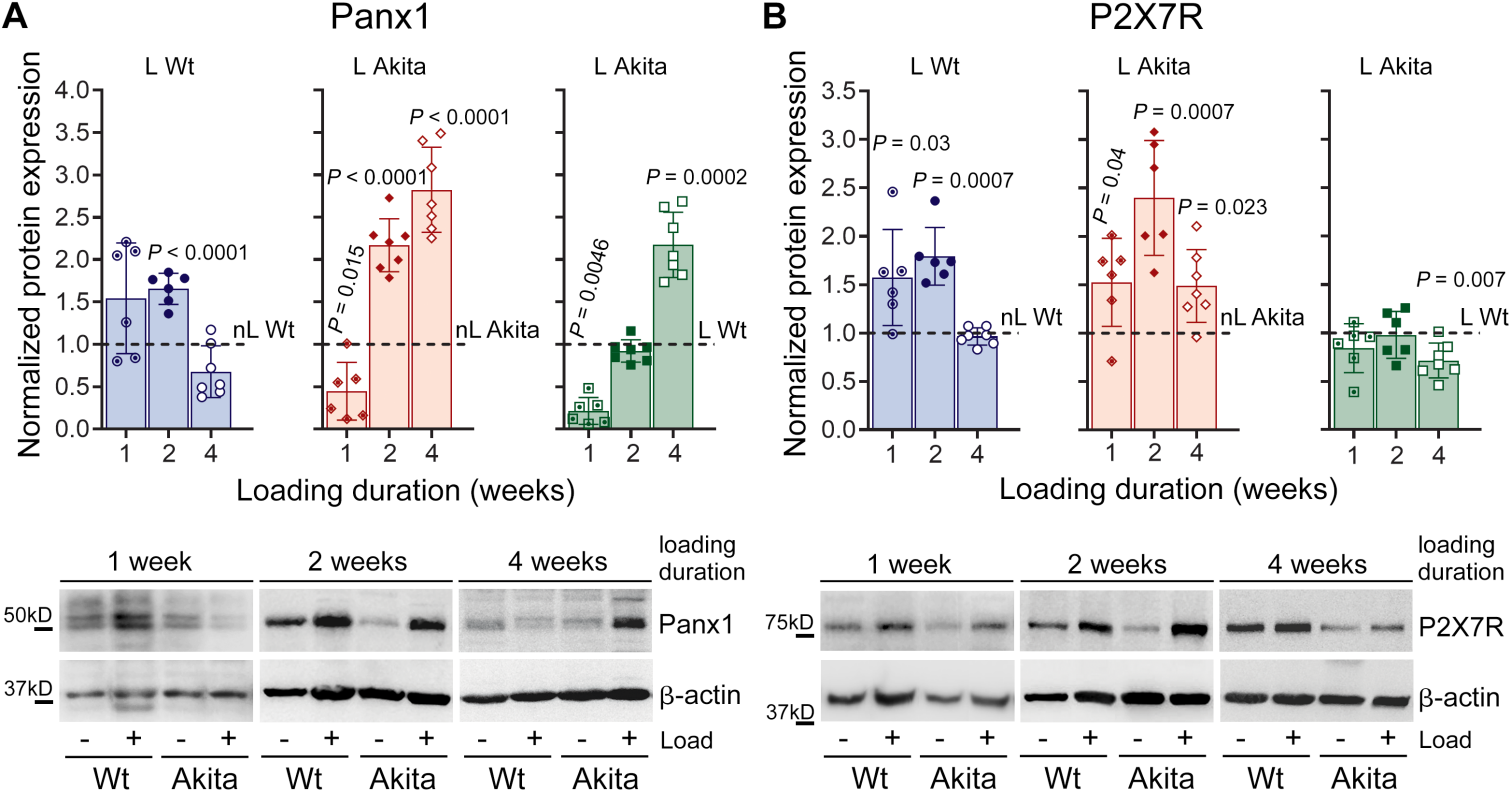
Panx1-P2X7R mechanosignaling complex responds differently to mechanical loading in wild-type (Wt) and T1D Akita mice. Relative protein expression of **(A)** Panx1 and **(B)** P2X7R in loaded (L) Wt and Akita mice compared to their age-matched non-loaded (nL) Wt after 1, 2 and 4 weeks of loading (treadmill running). Protein levels were first normalized with respective loading control, β-actin, followed by respective age-matched non-loaded controls (denoted as dotted lines) for Wt (blue bars) and Akita (red bars) or respective age-matched loaded Wt (green bars, denoted as dotted line). *P* values were obtained using an unpaired t-test followed by either Welch’s or Mann-Whitney test. Loading duration groups: n = 6 (1 week), n = 6 (2 weeks), n = 7 (4 weeks). Data presented as means ± SD.

We next submitted both Wt and T1D Akita young adult mice to 1, 2 and 4 weeks of treadmill running to investigate if mechanical loading alters the expression of this mechanosignaling complex and whether this response is altered in the diabetic bones. We observed adaptive, duration-dependent changes in Panx1 and P2X7R in response to mechanical loading in both Wt and T1D Akita mice, as shown in **Figure 4**. In Wt bone, after 1 week of loading, Panx1 expression showed a slight increase, whereas P2X7R was significantly upregulated. By 2 weeks, both Panx1 and P2X7R protein levels were modestly but significantly increased in loaded Wt bone when compared to their age-matched non-loaded controls (**Figure 4**, blue bars). By 4 weeks, expression of both proteins returned to levels comparable to those of non-loaded Wt bone, indicating a normalization and adaptation to the loading regime. Importantly, this adaptive Panx1-P2X7R response to mechanical loading was also present in the diabetic bone (**Figure 4**, red bars), although the temporal pattern differed. In Akita bone, Panx1 expression was significantly downregulated after 1 week of loading, while P2X7R was significantly upregulated. By 2 weeks, both Panx1 and P2X7R were markedly upregulated in loaded Akita bone, resembling the response observed in loaded Wt bone. However, unlike Wt bone, which normalized by 4 weeks, Panx1 and P2X7R levels remained significantly elevated in Akita bone when compared to their age-matched non-loaded Akita bone (**Figure 4**, red bars), indicating a prolonged or dysregulated response to mechanical loading. Further comparison between loaded Akita and loaded Wt bone revealed that the adaptive response of Panx1 and P2X7R was dysregulated in diabetic bone (**Figure 4**, green bars). After 1 week of loading, Panx1 expression was significantly reduced in loaded Akita bone, while P2X7R levels were comparable to loaded WT bone (**Figure 4**, green bars). By 2 weeks of loading, expression of both proteins in loaded Akita bone appeared similar to that observed in loaded Wt bone. However, after 4 weeks of loading, marked difference reemerged: Panx1 expression remained significantly elevated, whereas P2X7R expression was significantly reduced in loaded Akita bone when compared to age-matched loaded Wt bone. Together, these findings indicate that the temporal adaptation of Panx1 and P2X7R to mechanical loading is dysregulated in the diabetic bone.

**Figure 5:**
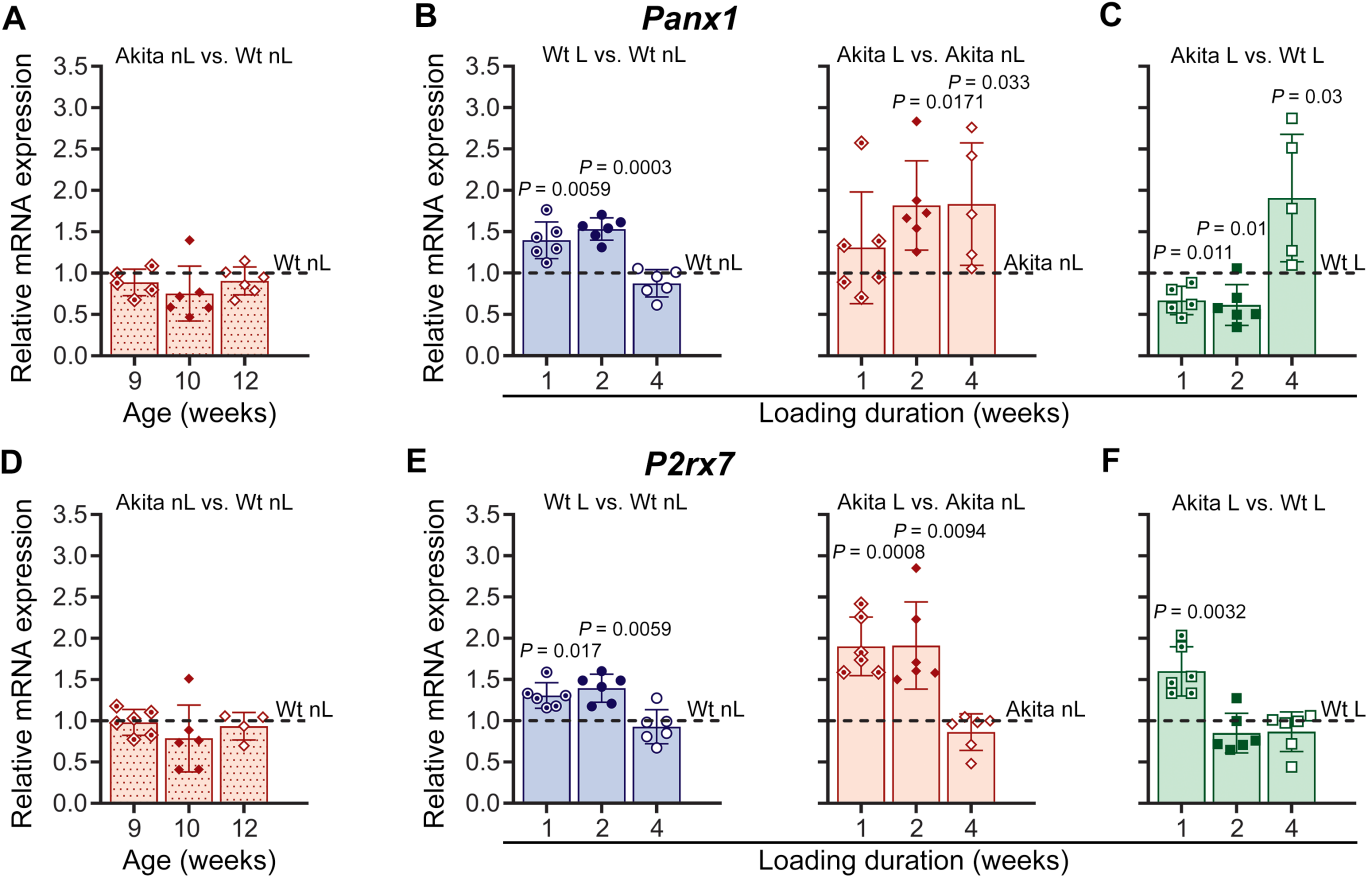
Mechanical loading induces different Panx1 and P2X7R transcriptional responses in wild-type (Wt) and Akita mice. Relative mRNA expression of Panx1 (*Panx1*) (top panel) and P2X7R (*P2rx7*) (lower panel). **(A, D)** Non-loaded (nL) Akita mice at 9, 10, and 12 weeks of age compared with age-matched Wt controls. **(B, E)** Akita and Wt mice after 1, 2, and 4 weeks of treadmill loading (L), comparing loaded versus respective age-matched non-loaded controls. **(C, F)** Loaded Akita mice compared with their respective age-matched loaded Wt. The delta-delta CT method was used for data analysis, where the value of each gene of interest is first normalized to the reference gene (18S) and then to either their age matched non-loaded Wt (dotted red bars, denoted as dotted lines) or their respective age matched non-loaded controls (denoted as dotted lines) for Wt (blue bars) and Akita (solid red bars) or respective age-matched loaded Wt (green bars). *P* values for comparisons between Wt nL vs. Akita nL, Wt L vs. Wt nL, Akita L vs. Akita nL, and Akita L vs. Wt L at each time point were obtained using an unpaired t-test followed by either Welch’s or Mann-Whitney test. Loading duration groups: n= 6 (1, 2 weeks), n = 5-7 (4 weeks). Data presented as means ± SD.

This effect of T1D on the bone adaptive response was also evidenced by subsequent data obtained from quantification and comparison of Panx1 and P2X7R mRNA expression at 1, 2, and 4 weeks of treadmill running. As shown in **Figure 5**, both *Panx1* and *P2rx7* expression levels in non-loaded Akita bone were relatively the same and comparable to those of non-loaded Wt bones (**Figures 5A, D**). However, when the mice were subjected to mechanical loading, both *Panx1* and *P2rx7* transcript levels in loaded bones, when compared to non-loaded Wt bones, were significantly upregulated as early as 1 week of loading, remained upregulated at 2 weeks of loading, and then returned to baseline levels at 4 weeks of loading (**Figures 5B, E**, solid blue bars). In the Akita bone, we observed a slightly different load-induced transcriptional response. In loaded Akita bones, a marked upregulation of *Panx1* mRNA expression relative to non-loaded Akita bones was also detected, but at 2-week loading duration, later than observed in loaded Wt bones, and remained upregulated after 4 weeks of loading (**Figure 5B**, solid red bars). In contrast, the pattern of load-induced changes in *P2rx7* expression did not differ between Akita and Wt loaded bones when compared to their respective age-matched counterparts (**Figure 5E**, solid blue and red bars). Interestingly, when we compared loaded Wt with loaded Akita bone, we noticed that load-induced *Panx1* activation in Akita mice was significantly lower than in Wt mice at 1 and 2 weeks and markedly higher after 4 weeks of loading (**Figure 5C**), whereas load-induced *P2rx7* activation was significantly higher in Akita after 1 week of loading (**Figure 5F**).

### 3.4 Load-induced inflammation in diabetic bones

**Figure 6:**
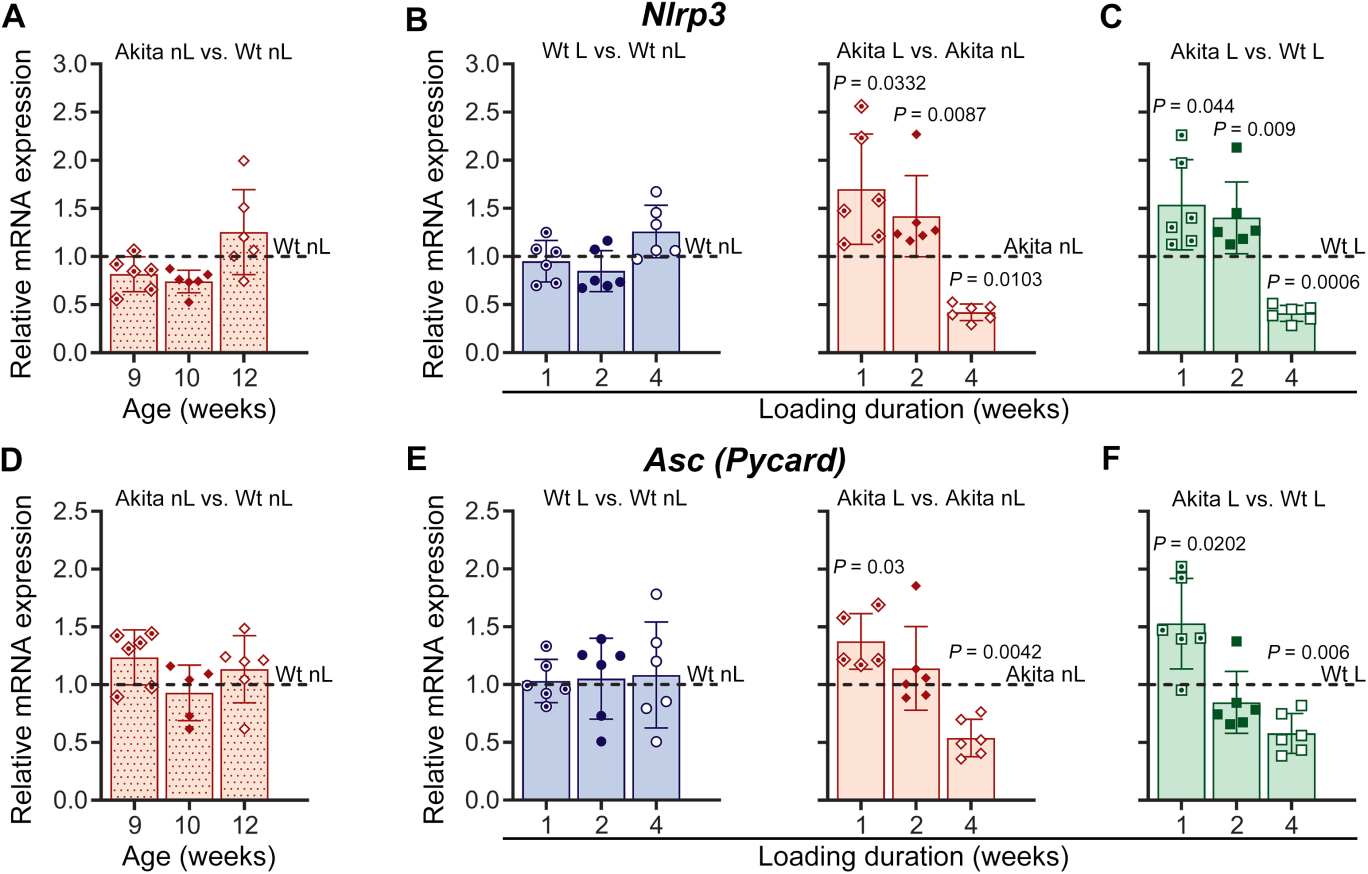
Loading activates NLRP3 inflammasome components in diabetic bone. Relative mRNA expression of NLRP3 (*Nlrp3*) (top panel) and ASC (*Asc/Pycard*) (lower panel). **(A, D)** Non-loaded (nL) Akita mice at 9, 10, and 12 weeks of age compared with age-matched Wt controls. **(B, E)** Akita and Wt mice after 1, 2, and 4 weeks of treadmill loading (L), comparing loaded versus respective age-matched non-loaded controls. **(C, F)** Loaded Akita mice compared with their respective age-matched loaded Wt. The delta-delta CT method was used for data analysis, where the value of each gene of interest is first normalized to the reference gene (18S) and then to either their age matched non-loaded Wt (dotted red bars, denoted as dotted lines) or their respective age matched non-loaded controls (denoted as dotted lines) for Wt (blue bars) and Akita (solid red bars) or respective age-matched loaded Wt (green bars). *P* values for comparisons between Akita L vs. Akita nL, and Akita L vs. Wt L at each time point were obtained using an unpaired t-test followed by either Welch’s or Mann-Whitney test. Loading duration group: n= 5-6 (1, 2, 4 weeks). Data presented as means ± SD.

**Figure 7:**
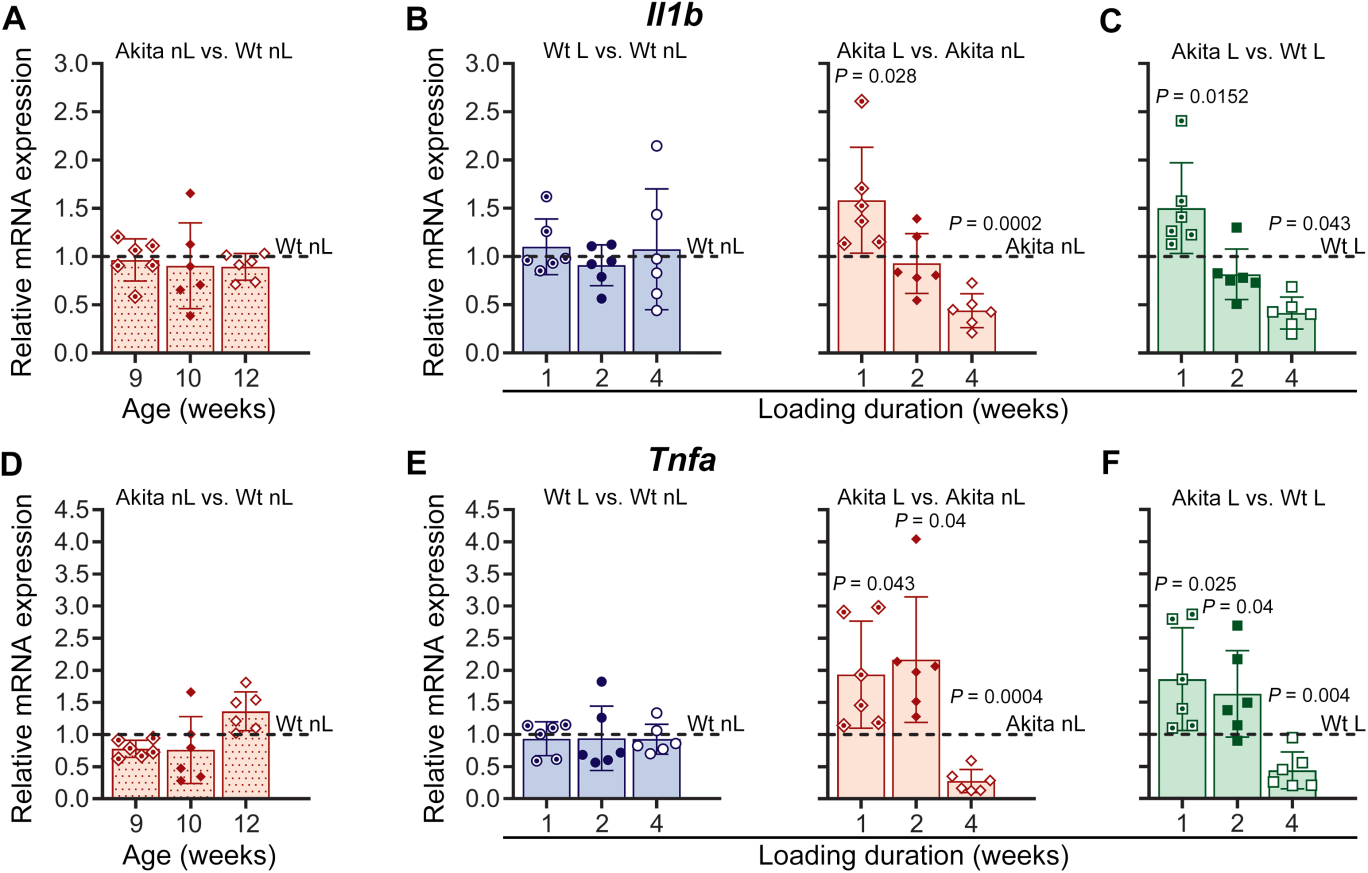
Loading upregulates inflammatory cytokines in diabetic bone. Relative mRNA expression of IL-1β (*Il1b*) (top panel) and TNF-α (*Tnfa*) (lower panel). **(A, D)** Non-loaded (nL) Akita mice at 9, 10, and 12 weeks of age compared with age-matched Wt controls. **(B, E)** Akita and Wt mice after 1, 2, and 4 weeks of treadmill loading (L), comparing loaded versus respective age-matched non-loaded controls. **(C, F)** Loaded Akita mice compared with their respective age-matched loaded Wt. The delta-delta CT method was used for data analysis, where the value of each gene of interest is first normalized to the reference gene (18S) and then to either their age matched non-loaded Wt (dotted red bars, denoted as dotted lines) or their respective age matched non-loaded controls (denoted as dotted lines) for Wt (blue bars) and Akita (solid red bars) or respective age-matched loaded Wt (green bars). *P* values for comparisons between Akita L vs. Akita nL, and Akita L vs. Wt L at each time point were obtained using an unpaired t-test followed by either Welch’s or Mann-Whitney test. Loading duration group: n = 6 (1, 2, 4 weeks). Data presented as means ± SD.

In addition to its role in mechanosignaling, the Panx1-P2X7R complex has also been implicated in inflammatory responses across various cell types by activating the NLRP3 inflammasome. Studies with STZ-induced T1D mice have shown that the levels of inflammatory cytokines within the local bone environment increase at early stages of diabetes and subsequently normalize with disease progression (37). This finding led to the proposal that local inflammatory responses may contribute to the mechanisms underlying diabetic osteopenia. Dysregulation of the Panx1-P2X7R complex could be one of the factors driving this local inflammatory response in diabetic bones. We quantified mRNA levels of inflammasome components and inflammatory cytokines in Wt and diabetic Akita non-loaded and loaded bones to assess the level of local inflammation and evaluated the impact of treadmill running on these responses. Our data demonstrate that under non-loaded conditions, the transcript levels of the NLRP3 inflammasome components *Nlrp3* and *Asc* (**Figures 6A, D**) as well as *Casp1* (data not shown), were not significantly different in Akita bone compared to Wt bone. Transcript levels of the inflammatory *Il1b* and *Tnfa* were also not different in non-loaded Akita and Wt bones (**Figures 7A, D**). However, we observed that when the bones were challenged with 1 to 4 weeks of treadmill running, loading induced an inflammatory response in T1D Akita bone (**Figures 6B, E**, **7B, E**, solid red bars) that was absent in Wt bone (**Figures 6B, E**, **7B, E**, blue bars). As shown in **Figures 6**, **7** (B, E, solid red bars), mechanical loading significantly upregulated expression levels of *Nlrp3*, *Asc*, *Il1b*, and *Tnfa* in Akita bone after 1 week of loading when compared to non-loaded Akita bone. *Nlrp3*, *Tnfa* expression remained elevated in loaded Akita bone, while *Asc* and *Il1b* levels normalized to those of non-loaded Akita bone after 2 weeks of loading. By 4 weeks, all the inflammation markers mentioned above exhibited a significant downregulation in loaded Akita bone. This effect of loading on diabetic bones is further evidenced when comparing loaded Akita and loaded Wt bones, showing a marked load-induced temporal regulation of the NLRP3 inflammasome (*Nlrp3* and *Asc/Pycard*) and inflammation mediators in loaded diabetic bones (**Figures 6C, F**, **7C, F**). *Casp1* levels remained unaltered throughout the 4-week loading in both Akita and Wt bones (data not shown).

### 3.5 Effects of high glucose on load-induced regulation of the mechanosignaling complex and activation of inflammatory responses in osteocytes

**Figure 8:**
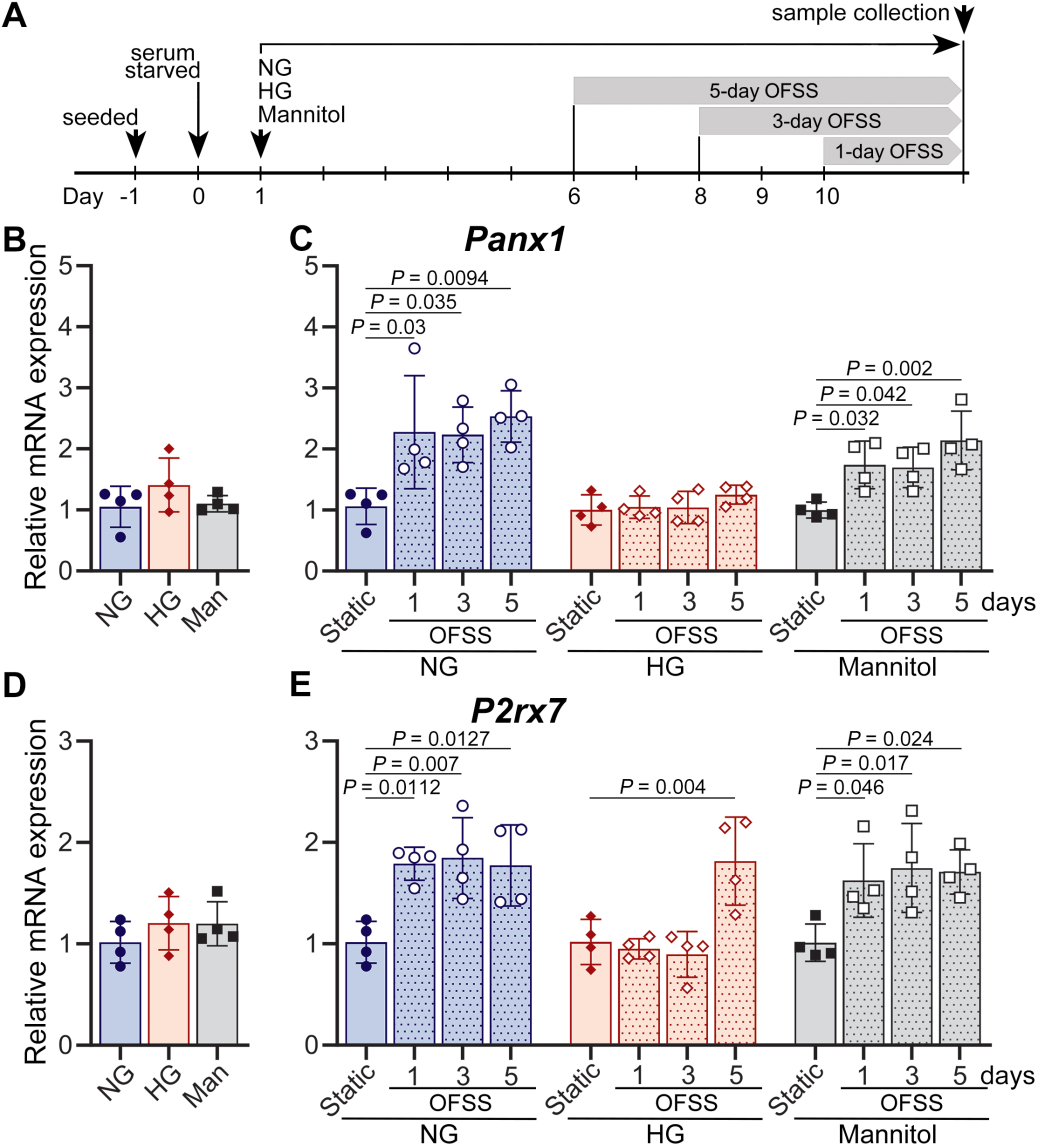
Exposure to high glucose blunts oscillatory shear stress (OFSS)-induced Panx1 and P2X7R mRNA expression in MLO-Y4 cells. **(A)** Experimental scheme showing the duration of normal glucose (NG), high glucose (HG), and mannitol (Man) exposure, indicating the points at which 1, 3, and 5 days of 30 minutes OFSS in MLO-Y4 cells were initiated, and sample collection at day 10. Relative mRNA expression of Panx1 (*Panx1*) and P2X7R (*P2rx7*) under **(B, D)** static conditions and **(C, E)** after 1, 3, and 5 days of OFSS in MLO-Y4 cells conditioned under NG, HG, and mannitol. The delta-delta CT method was used for data analysis, where the value of each gene of interest is first normalized to the reference gene (18S) and then to static NG **(B, D)** or respective static NG, HG, or Man **(C, E)**. *P* values for comparisons among various conditions were obtained using one-way ANOVA followed by Dunnett’s multiple comparisons test. n= 4/condition. Data presented as means ± SD.

We reported that *in vitro* exposure to HG significantly alters the Panx1-P2X7R protein expression levels in MLO-Y4 osteocytes (21). We have now investigated the extent to which loading regulates the expression of this mechanosignaling complex in osteocytes by mimicking 1 week of *in vivo* loading under HG levels normally associated with diabetes. Experiments were set up as shown in **Figure 8A**. We found that after 10 days of exposure to HG or to Man, the *Panx1* and *P2rx7* mRNA expression levels were not different from those measured from cells exposed to NG (**Figures 8B, D**). Loading imposed by daily 30 minutes of OFSS resulted in marked upregulation of *Panx1* and *P2rx7* mRNA expression levels in MLO-Y4 cells cultured under NG and in Man when compared to respective static conditions. This response was observed as early as 1 day of OFSS and was maintained at similar higher levels after 3- and 5-day exposure to OFSS (**Figures 8C, E**; dotted blue and gray bars). In contrast, under HG, *Panx1* expression levels in osteocytes were unaffected by OFSS (at all three time points) when compared to those of respective static controls (**Figure 8C**, dotted red bars). *P2rx7* expression levels, on the other hand, were significantly upregulated under HG but only after 5-day exposure to OFSS (**Figure 8E**, dotted red bars). When we subsequently compared OFSS-induced changes in *Panx1* and *P2rx7* mRNA expression levels under all three conditions, no major differences between NG and Man treatments were observed, while considerable downregulation of the mechanosignaling complex was detected under HG: *Panx1* at all three time points, and *P2rx7* at 1 and 3 days (**Supplementary Figure 2**).

**Figure 9:**
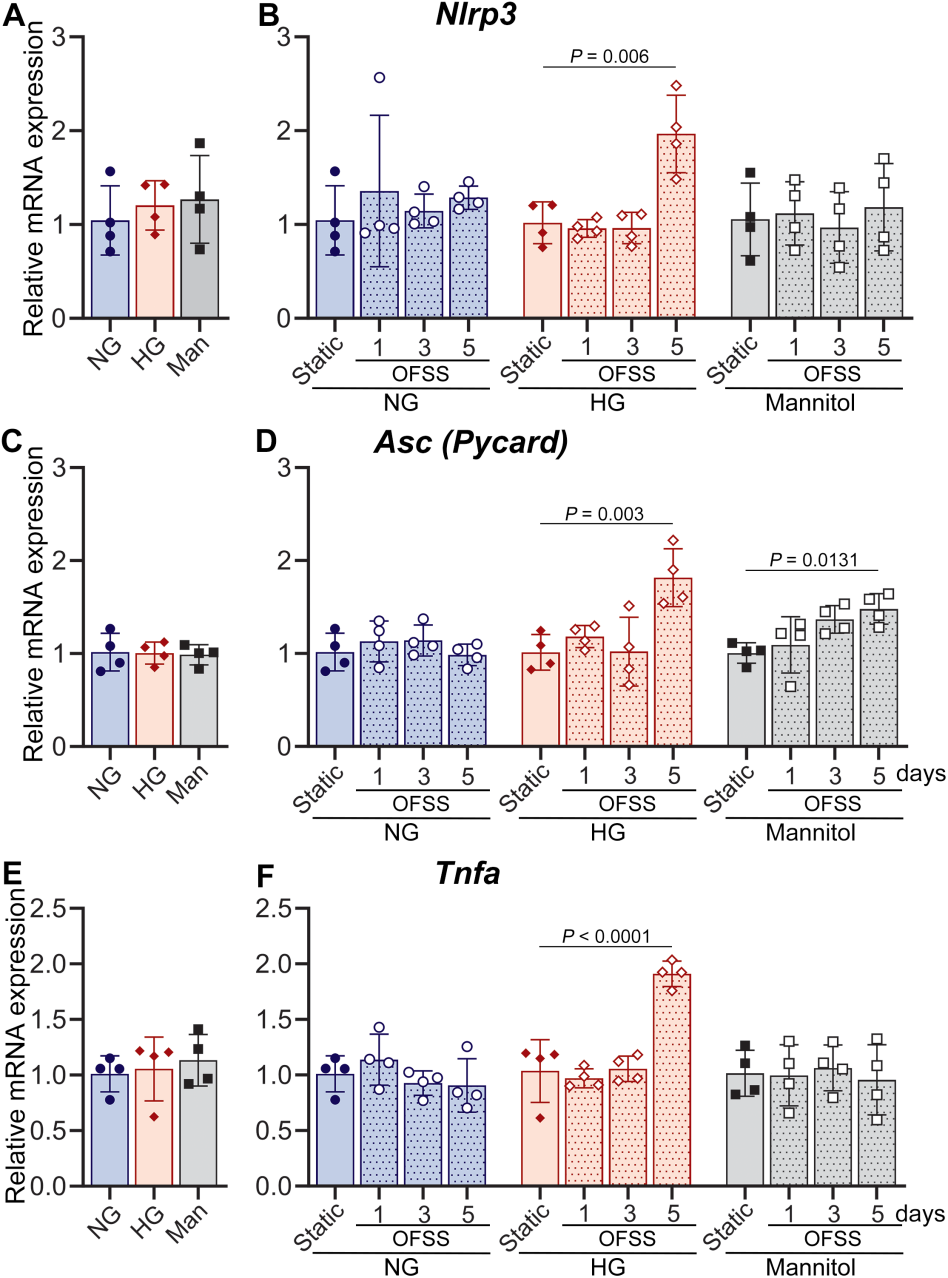
Exposure to high glucose upregulates oscillatory shear stress (OFSS)-induced transcription of NLRP3 inflammasome components and inflammatory cytokines in MLO-Y4 cells. Relative mRNA expression of NLRP3 (*Nlrp3*), ASC (*Asc, Pycard*) and TNF-α (*Tnfa*) under **(A, C, E)** static condition and **(B, D, F)** after 1, 3, and 5 days of 30 minutes OFSS in MLO-Y4 cells conditioned in normal glucose (NG), high glucose (HG) and mannitol (Man). The delta-delta CT method was used for data analysis, where the value of each gene of interest is first normalized to the reference gene (18S) and then either to static NG **(A, C, E)** or respective static NG, HG, or Man **(B, D, F)**. *P* values for comparisons among various conditions were obtained using one-way ANOVA followed by Dunnett’s multiple comparisons test. n= 4/condition. Data presented as means ± SD.

Given the aforementioned role of the Panx1-P2X7R complex in inflammatory responses involving NLRP3 inflammasome activation (28), which results in caspase-1 activation and the release of pro-inflammatory cytokines IL-1β and IL-18 (29, 30), we next examined whether the HG-induced impairment of load-regulated mechanosignaling was accompanied by activation of inflammatory responses. We examined mRNA levels of inflammasome components (*Nlrp3, Asc, Casp1*) and inflammatory cytokines (*Il1b, Tnfa*) in MLO-Y4 cells exposed to OFSS. Our data showed that under static NG, HG, Man conditions, NLPR3 inflammasome component and inflammatory cytokines levels are relatively the same (**Figures 9A, C, E**). Similar to what was observed in Wt bone after 1 week of *in vivo* loading, *in vitro* exposure of MLO-Y4 cells to OFSS under NG conditions did not induce an inflammatory response when compared to the respective static controls (**Figures 9B, D, F**, dotted blue bars). Likewise, no notable OFSS-induced inflammation was detected under Man conditions when compared to the respective static controls, except for the upregulation of *Asc* at 5-day exposure of OFSS (**Figures 9B, D, F**, dotted gray bars), which likely indicates an effect of osmolarity in this inflammasome component. In contrast, under HG conditions, the expression levels of not only *Asc* but also *Nlrp3* and the inflammatory cytokine *Tnfa* were markedly upregulated after 5 days of exposure to OFSS when compared to the respective static controls, suggesting activation of an inflammatory response (**Figures 9B, D, F**, dotted red bars). Further comparison of OFSS-induced expression of inflammation markers under HG and Man conditions with that of the NG condition confirmed the activation of OFSS-induced inflammation exclusively under the HG condition (**Supplementary Figure 3**, dotted red bars). Note: *Casp1* mRNA expression remained unaltered in MLO-Y4 osteocytes under all conditions examined (data not shown).

## 4 Discussion

Skeletal health is adversely affected by diabetes (5, 7). The link between T1D, reduced bone mass, and increased risk of fractures has been well established. However, the precise mechanisms responsible for diabetic osteopenia remain incompletely understood. Studies with rodent models of T1D have been instrumental in gaining insight into these mechanisms. One caveat is that the majority of these studies report on findings from the adult skeleton (9, 10, 14, 17, 18, 37), with very few focusing on the young adult skeleton (16, 57). Individuals with juvenile-onset T1D are exposed to the disease for a longer duration in their lifetime and are consequently at risk for developing diabetic complications earlier in life (58). There is evidence that, similar to T1D adults, T1D children and young adults are at increased risk of fracture (1–5, 7). Several studies have reported a higher prevalence of reduced bone mass, including osteopenia, in children and adolescents (7–14 years of age) with T1D compared to their non-diabetic counterparts soon after diagnosis (2, 3). Considering that peak bone accrual occurs in adolescence in concurrence with the pubertal growth spurt, this critical phase may represent not only an interval of increased skeletal vulnerability, but also a window of opportunity for therapeutic intervention (6).

In this study, we addressed this gap in our knowledge by characterizing changes at the tissue level and exploring cellular and molecular mechanisms that may be implicated in bone dysfunction associated with early-onset diabetes in young adult T1D Akita mice. We placed particular emphasis on the osteocyte responses to mechanical loading to test the overarching hypothesis that dysregulation of the osteocyte Panx1-P2X7R mechanosignaling complex in diabetic bone disrupts proper load-induced bone adaptation and mediates load-induced local inflammatory responses via inflammasome activation that contribute to bone loss in T1D.

Previous studies have shown that 10-week-old T1D Akita mice display substantially lower bone mineral density (BMD) and distal femur bone volume with accompanying bone loss (16), and that older adult Akita mice (~ 21 weeks) display impaired fracture healing (59), bone strength, and quality (60). Data reported herein from our longitudinal studies conducted with T1D Akita mice starting a 5 weeks of age have now demonstrated that the deleterious effects of diabetes on the skeleton start early, with significant reduction in bone density that is observed as soon as 8 weeks of age and remains throughout adulthood (approximately from 12 to 28 weeks of age) (**Figure 1A**). These findings from T1D Akita mice are consistent with clinical studies reporting the impact of diabetes on the developing skeleton. Children and adolescents (7–14 years of age) with T1D display a higher prevalence of reduced bone mass, including osteopenia, when compared to their non-diabetic counterparts soon after diagnosis (2, 3). Studies using 3D quantitative computed tomography have shown a significant reduction in cortical volumetric BMD (vBMD) and a higher buckling ratio (a marker for cortical instability) in the intertrochanteric region of young to middle-aged T1D men when compared to non-diabetic controls (61). Impaired bone health in young adults with T1D, indicated by marked changes in trabecular and cortical microarchitectural values, was recently reported in studies using high-resolution peripheral quantitative computed tomography (HR-pQCT) (62). Furthermore, it has been noted that children or adolescents with T1D have a smaller cortical area, bone mass, and bone size compared to healthy young adults, and this deficit persists over time during their pubertal development (62–64). Likewise, our observation of significantly smaller bone parameters (Ct.B.Ar, Ct.B.Ar/Tt.Ar, Ct.Th, *J, Ix, Iy*) in young adult Akita mice (12 weeks of age) together with Parajuli et al. (18) findings of smaller bone parameters in older Akita mice (~ 32 weeks of age) when compared to their age-matched Wt counterparts, strongly indicate that skeletal health is greatly compromised soon after the onset of T1D. Therefore, these findings collectively establish that T1D affects skeletal integrity during early adolescence and continues to have sustained impacts throughout adulthood. These deleterious effects of T1D have the potential to lead to significant complications in skeletal health, given that cortical bone is a key component of bone tissue in the skeleton, and disruptions in its integrity can increase fracture risk.

Skeletal health relies on the bone’s ability to respond and adapt to mechanical stimuli. While it has been reported that the bone response to mechanical loading is significantly impaired in the adult male T1D mouse skeleton (17, 18), there has been relatively limited exploration in the context of adolescent and young adult skeletons. Findings described herein revealed that load-induced anabolic response is completely abolished in adolescent and young adult Akita male mice subjected to treadmill running (**Table 1**; **Figure 2**). The characteristic robust load-induced bone formation, in particular periosteal bone appositions in hindlimbs, normally detected in age-matched Wt mice (26, 42) is absent in both young and adult Akita males (**Figures 2C–E**, red bars). Similar lack of anabolic response has been reported in studies with adult Akita and STZ male mice subjected to ulna loading (17, 18). These findings further demonstrate the inability of the diabetic bone to adapt to mechanical loading. Our observation of no apparent osteocyte apoptosis in loaded young adult Akita bone (**Supplementary Figure 1**), unlike previous reports in adult T1D bone (17), suggests that the osteocyte apoptosis is not a factor contributing to the impaired load-induced adaptation during early life-onset diabetes. Findings reported herein indicate that one of the factors is likely the inability of the diabetic bone to properly sense and respond to mechanical stimulation due to an impaired load-induced regulation of the osteocyte Panx1-P2X7R complex.

We have shown that the mechanosensitive Panx1 and the purinergic P2X7R form a functional complex in osteocytes that plays a key role in osteocyte mechanosignaling by providing a major pathway for flow-induced ATP release (21). The critical roles of Panx1 and P2X7R in load-induced bone adaptation have been established in previous *in vivo* and *in vitro* studies by us and others (21, 25, 26). Findings from this current study showing that the decrease in cortical bone integrity observed in young adult T1D Akita mice (**Figure 1**, **Table 1**) is accompanied by a significantly lower Panx1 and P2X7R protein expression levels (**Figure 3**) implicate the Panx1-P2X7R complex in the deleterious effects of diabetes on the bone and further highlights its importance for proper skeletal health.

In this study, we have also demonstrated that mechanical loading regulates the expression of both Panx1 and P2X7R in a bi-phasic manner in osteocyte-enriched Wt bones. Our data show that load-induced anabolic responses in young adult Wt bones are accompanied by adaptive upregulation of Panx1-P2X7R expression at the 1- and 2-week loading phase, follow by normalization of expression at the 4-week loading phase (**Figure 4**, blue bars) indicating that full adaptation with robust anabolic response is achieved (**Table 1**; **Figure 2**). Similar findings were previously reported by us for young adult bones of a different mouse strain (C57Bl/6NCrl) (26). Load-induced adaptive upregulation of *Panx1* and *P2rx7* mRNA expression is apparent as early as 1 week, remains elevated at 2 weeks, and is normalized at 4 weeks of loading (**Figures 5B, E**, blue bars), suggesting that transcriptional load adaptation takes place during the first 2 weeks of loading in Wt bones. Further *in vitro* assessments reveal that OFSS-induced adaptive response in osteocytes at 1, 3, and 5 days under control NG conditions is not only comparable to that of an *in vivo* 1-week loading bout but also begins immediately after 1 day of loading (**Figures 8C, E**, blue dotted bars).

Notably, this manner of Panx1-P2X7R response to mechanical loading is altered in diabetic bones. At the protein level, this is evident as early as 1 week of loading, where an asynchronous response is observed, characterized by reduced Panx1 expression and a concomitant increase in P2X7R (**Figure 4**, red bars). By 2 weeks of loading, both Panx1 and P2X7R are upregulated, resembling the adaptive response observed in Wt bone. However, unlike Wt bone, Panx1 and P2X7R expression levels remain elevated after 4 weeks of loading (**Figure 4**, red bars). A similar pattern is observed at the transcriptional level. *Panx1* and *P2rx7* mRNA expression levels are upregulated during the adaptation phase, 1–2 weeks of loading (**Figures 5B, E**, red bars). However, by 4 weeks, mRNA expression in diabetic bone becomes dysregulated, in contrast to the normalization observed in WT bone. Direct comparisons between Wt-loaded and Akita-loaded bones further highlight this disruption. Although Panx1 and P2X7R protein levels in Akita bone become comparable to Wt levels during the adaptive phase (2 weeks), this occurs following an initial asynchronous response at 1 week. By 4 weeks, expression diverges again and becomes dysregulated in diabetic bone (**Figure 4**, green bars). This dysregulation is even more pronounced at the mRNA level, where disruption of *Panx1* and *P2rx7* expression is evident as early as 1 week of loading, and the coordinated regulation observed in Wt bone is lost throughout the 4-week loading period (**Figures 5C, F**). These findings are in line with our *in vitro* analysis, where OFFS-induced adaptive response in osteocytes under HG conditions is initially absent (1 and 3 days of OFSS) and observed only for the *P2rx7* at 5 days of OFSS (**Figures 8C, E**, red dotted bars), similar to the response observed in the bones at 1-week *in vivo* loading. Taken together, these findings strongly suggest that the young adult diabetic bone responds but is unable to adapt to mechanical loading. This conclusion is supported by the observation that a higher magnitude of strain (2000-3500 μϵ) is required to induce periosteal bone formation in adult diabetic ulna, implying that the diabetic bone is unable to properly respond to physiological loading (17, 18). Mechanical loading imposed by treadmill running in our study is complex and multiaxial, unlike tibial or ulnar loading, which is a direct uniaxial load. Given that in 12-week-old mice i) low strain levels are associated with habitual activities (walking: < 200 µϵ of tension, jumping: < 600 µϵ of compression) and ii) over 1000 µϵ is required for an anabolic response to tibial loading (65), the robust anabolic response to treadmill running observed in Wt bones by us and others (26, 42) collectively suggest that the strain level experienced during treadmill running in femurs is likely on the order of ~ 1000 μϵ. Therefore, the lack of anabolic response in diabetic mice seen in this study is partly due to low engendered strain level. Overall, under physiological settings, our current findings support the notion that osteocytes, considering their well-known role as mechanosensing cells, are incapable of properly detecting the mechanical stimuli under diabetic conditions.

Our observation of upregulated Panx1 and P2X7R expressions in young adult diabetic bones at the end of the 4-week loading period, particularly for Panx1 at both protein and gene expression levels, indicates their participation in mechanisms of load-induced bone adaptation that may extend beyond their role as an osteocyte Panx1-P2X7R mechanosignaling complex. Both Panx1 and P2X7R are implicated in NLRP3 inflammasome activation and IL-1β release in neurons, astrocytes, cancer cells, and immune cells (28–34). Given that inflammatory conditions are known to be associated with bone loss and T1D (35–38), we further investigate whether altered Panx1 and P2X7R could be implicated in the activation of inflammatory responses in osteocytes. Our data shows that local NLRP3 inflammasome involvement (as indicated by upregulation of *Nlrp3* and *Asc*) and elevated proinflammatory cytokines expression (upregulation of *Il1b* and *Tnfa*) are evident in diabetic bone as early as 1 week of loading (**Figures 6B, C, E, F**, **7B, C, E, F**, solid red bars and green bars). This finding is supported by our *in vitro* observation demonstrating the upregulation of NLRP3 inflammasome and the proinflammatory cytokine *Tnfa* in osteocytes under HG conditions after 5 days of OFSS (**Figures 9B, D, F**, red dotted bars). These results, showing that both *in vitro* and *in vivo* load-induced upregulation of *P2rx7* expression in osteocytes under diabetic conditions, either after 5 days of OFSS or 1 week of treadmill running concur with the observed increase of inflammasome components and proinflammatory cytokines (**Figures 5**-**9**, solid red and red dotted bars and **Supplementary Figure 2**) strongly suggest the involvement of P2X7R is this initial inflammatory response. Moreover, load-induced inflammation appears to be exacerbated (sustained upregulation of *Nlrp3* and *Tnfa*) in the diabetic bone during the adaptive loading phase (1–2 weeks of loading), which is the critical time window where a robust anabolic response is initiated in Wt bone. Significant reduction of NLRP3 inflammasome and proinflammatory cytokines expression (**Figures 6**, **7**) after 4 weeks of loading further indicates that load-induced flaring of inflammation is episodic and likely recurring with loading bouts. The lack of change in *Casp1* expression we observed in our experiments is consistent with prior reports showing that NLRP3 inflammasome signaling primarily regulates caspase-1 activation through proteolytic cleavage rather than transcriptional upregulation (66–69). Thus, inflammasome activity can be enhanced without altering total caspase-1 levels. This finding, together with the upregulation of both protein and mRNA expression of Panx1 and P2X7R in Akita bones at the 2-week loading time point, further suggests that excess levels of Panx1 and P2X7R are likely participating in the flaring of load-induced inflammation. The fact that both Panx1 protein and gene expression are still significantly elevated after 4 weeks of loading in T1D bones shows its involvement in the sustained inflammatory responses observed in diabetic bones, particularly in the latter phase of mechanical loading. It has to be noted that despite the reduction in inflammatory markers at 4 weeks of loading, the mechanoadaptive response in T1D bone remains impaired and does not show evidence of recovery (**Table 1**; **Figure 2**), suggesting that resolution of inflammation alone is insufficient to restore normal anabolic responsiveness. In this context, the persistent elevation of Panx1 and P2X7R protein expression, together with dysregulated gene expression, indicates continued disruption of mechanotransduction pathways despite attenuation of inflammasome activity.

**Figure 10:**
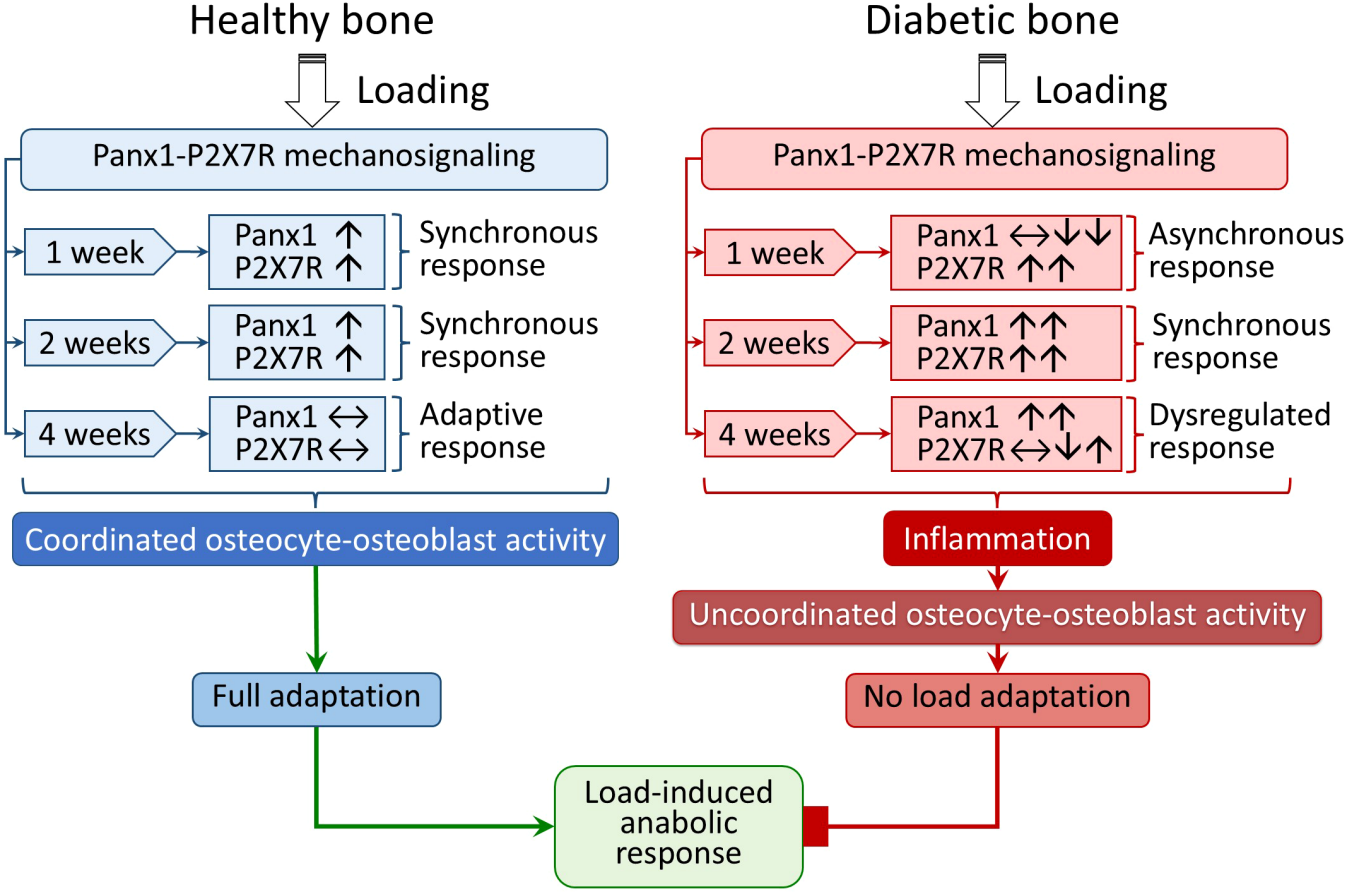
Summary of events driving the load-induced adaptive response in young adult T1D bone compared to healthy bone. The Panx1-P2X7R-mediated mechanosignaling in healthy bone (blue arrows: load adaptation pathways and green arrows: fully adapted pathways) shows a synchronous and adaptive response leading to a robust load-induced anabolic response, while in diabetic bone (red arrows), a dysregulated response together with activation of inflammation and possible recurrence of inflammation that leads to an impaired load-induced anabolic response. Arrows within the boxes represent transcriptional and protein-level changes at each time point.

Collectively, the sequence of events governing the load-induced adaptive response in young adult T1D can be summarized as illustrated in **Figure 10**. The scenario of Panx1-P2X7R involvement in the impaired load-induced ATP signaling under HG conditions that we proposed in our previous studies applies primarily to the onset phase of mechanical loading (first few days of loading). In the diabetic bone, this impaired response is accompanied by increased P2X7R expression and induction of inflammatory markers after 1 week of loading, followed by a subsequent rise in Panx1 levels after 2 weeks of loading (**Figure 10**, red arrows under diabetic bone). At this stage, the diabetic bone appears to “catch-up” and exhibit a coordinated response that is typically observed in Wt bone. However, the absence of an adaptive anabolic response after 2 weeks of loading and persistent Panx1-P2X7R upregulation suggests their involvement in sustaining the observed local load-induced inflammatory response in diabetic bone. In this context, abnormally higher extracellular ATP release may likely be observed locally as a result of augmented Panx1-P2X7R-mediated ATP-induced ATP release (21, 27) that may further intensify downstream signaling and enhance the release of load-induced proinflammatory cytokines, such as TNF-α, from osteocytes. This, in turn, could influence osteoblast function (37, 38, 70). Given that TNF-α can also regulate the NLRP3 inflammasome (45), this load-induced inflammatory signaling cascade could be further potentiated during 2 to 4 weeks of loading (**Figure 10**). Locally elevated cytokine gene expression in osteocytes may have an even greater impact on bone health because cytokine concentrations are likely highest at sites of secretion, thereby impairing osteocyte mechanosensing and responsiveness to mechanical loading, ultimately leading to compromised adaptation and bone loss associated with T1D.

In summary, this study demonstrates the adverse effects of T1D on physiological load-induced bone adaptation and skeletal health during a critical phase of skeletal growth that coincides with the early onset of juvenile diabetes. More importantly, we report for the first time a temporal disruption in the load-dependent regulation of Panx1 and P2X7R in young adult diabetic femurs, which we propose contributes to mechanisms underlying diabetic osteopenia. Our findings show that T1D not only compromises skeletal integrity but also impairs the adaptive response to mechanical loading, beginning in early adolescence and persisting into adulthood. This impairment appears to arise from dysregulation of the Panx1-P2X7R mechanosignaling complex, together with load-induced amplification of local inflammatory responses in diabetic bone, ultimately contributing to bone loss. Moreover, our results define a critical window during skeletal development during which bone health becomes particularly vulnerable following the onset of T1D. Collectively, these findings uncover additional diabetes-related mechanisms and advance our understanding of the factors that exert profound and long-lasting effects on skeletal health. Future studies, such as those employing targeted genetic and pharmacological approaches, will be important to gain further mechanistic insights into the link and role of Panx1-P2X7R signaling and inflammasome activation in diabetic bone.

## Data Availability

The raw data supporting the conclusions of this article will be made available by the authors, without undue reservation.
