# Supplementary material for: Panx1 and P2X7R are associated with impaired skeletal health and delayed bone development in early onset of type 1 diabetes
Source: Front Endocrinol (Lausanne). 2026 May 8;17:1829463. doi: 10.3389/fendo.2026.1829463 (PMC13194057; doi:10.3389/fendo.2026.1829463)

Supplemental data

Supplemental Table 1: Sequences of primers used for real-time qPCR

Gene	Species	Sequence	Main Text Reference
Panx1	mouse	Forward TATTGCCGTGGGTCTACCTC<br>Reverse TGTCGCCAGGAGAAAGAACT	(68)
P2rx7	mouse	Forward AAGCTGTACCAGCGGAAAGA<br>Reverse CCCACCCTCTGTGACATTCT	in house
Nlrp3	mouse	Forward ATTACCCGCCCGAGAAAGG<br>Reverse TCGCAGCAAAGATCCACACAG	(67)
Asc (Pycard)	mouse	Forward ACAGTACCAGGCAGTTCGTG<br>Reverse CAGGTCAGGTTCCAGGATGG	in house
Casp1	mouse	Forward GCTTCAATCAGCTCCATCAGC<br>Reverse GACGTGTACGAGTGGTTGTATTCA	in house
Il1b	mouse	Forward CAGGCAGGCAGTATCACTCA<br>Reverse TGTCCTCATCCTGGAAGGTC	(69)
Tnfa	mouse	Forward ACGGCATGGATCTCAAAGAC<br>Reverse GTGGGTGAGGAGCACGTAGT	(69)
18S	mouse	Forward CACGGCCGGTACAGTGAAAC<br>Reverse AGAGGAGCGAGCGACCAAA	(70)

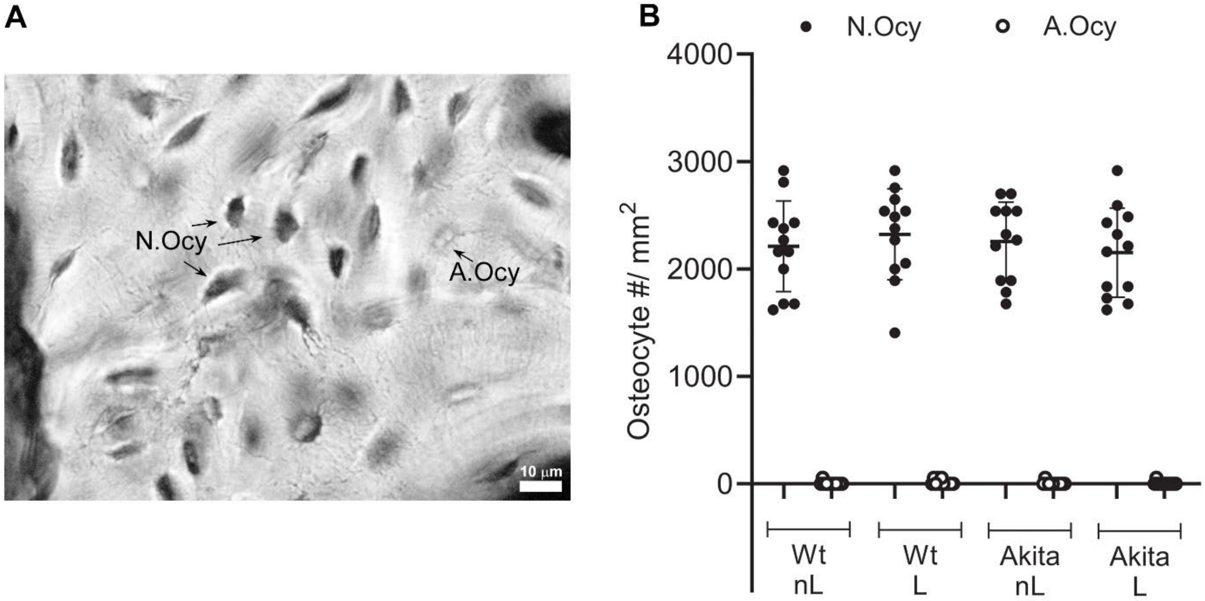

Supplemental Figure 1. (A) A representative phase image from the mid-shaft femur of a young adult wild-type (Wt) mouse showing examples of osteocyte integrity as assessed from PMMA-embedded basic fuchsin-stained ground sections after 4 weeks of loading. Normal osteocytes (N.Ocy) fully occupy lacunae, while atypical osteocytes (A.Ocy) appear retracted or pyknotic. Scale bar: 10 µm. (B) Osteocyte integrity (N.Ocy#/mm^2 versus A.Ocy#/mm^2) in Wt and Akita non-loaded and loaded mid-shaft femurs. Comparisons among groups (Wt and Akita non-loaded (nL), loaded (L)) were obtained using a one-way ANOVA test. n = 4 / group. Data presented as means ± SD.

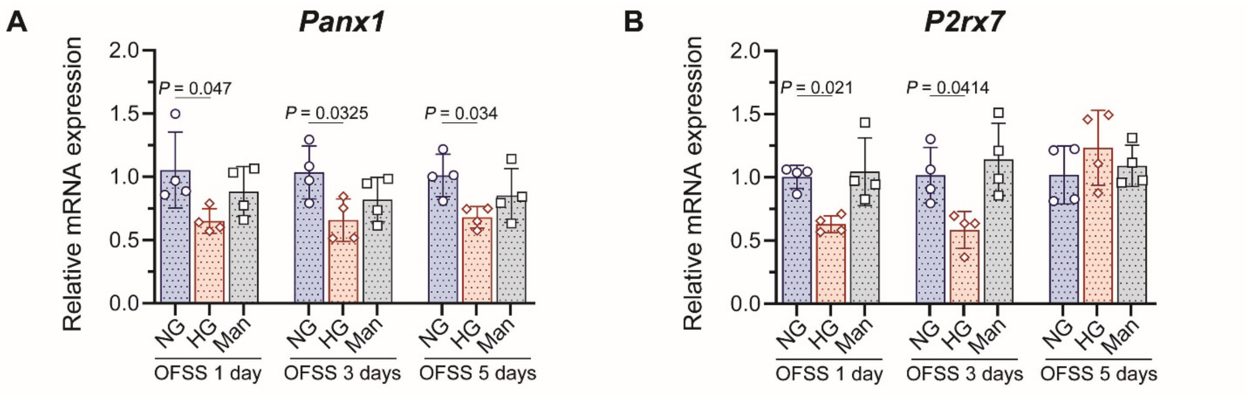

Supplemental Figure 2. Exposure to high glucose alters oscillatory shear stress (OFSS)-induced Panx1 and P2X7R mRNA expression in MLO-Y4 cells. Relative mRNA expression of (A) *Panx1* (Panx1) and (B) *P2rx7* (P2X7R) after 1, 3, and 5 days of 30 minutes OFSS in MLO-Y4 cells conditioned in normal glucose (NG), high glucose (HG), and mannitol (Man) with respect to respective OFSS-NG at each time point. *P* values for comparisons among various conditions were obtained using one-way ANOVA followed by Dunnett's multiple comparisons test. n= 4/condition. Data presented as means ± SD.

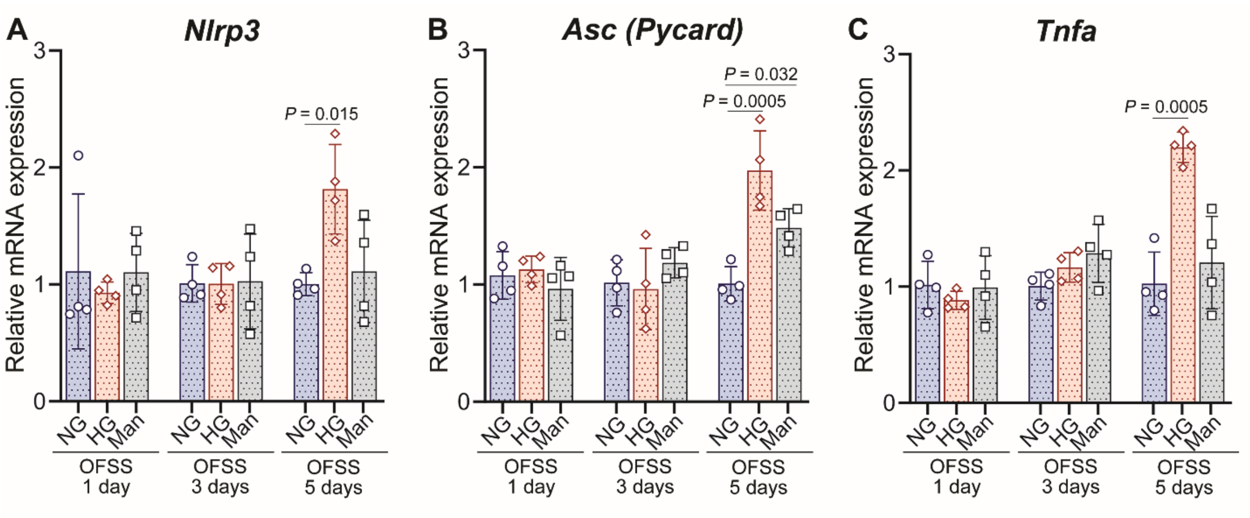

Supplemental Figure 3. Exposure to high glucose alters oscillatory shear stress (OFSS)-induced Panx1 and P2X7R mRNA expression in MLO-Y4 cells. Relative mRNA expression of (A) NLRP3 (*Nlrp3*), (B) ASC (*Asc, Pycard*) and (C) TNF-α (*Tnfa*) after 1, 3, and 5 days of 30 minutes OFSS in MLO-Y4 cells conditioned in normal glucose (NG), high glucose (HG) and mannitol (Man) with respect to respective OFSS-NG at each time point. *P* values for comparisons among various conditions were obtained using one-way ANOVA followed by Dunnett's multiple comparisons test. n= 4/condition. Data presented as means ± SD

Raw Western blot images

Figure 3

X: Denotes lanes not used as representative blots in the figure
nL: non-loaded samples, L: loaded samples

8W Panx1, P2X7R, and the corresponding β-actin loading controls

Panx1

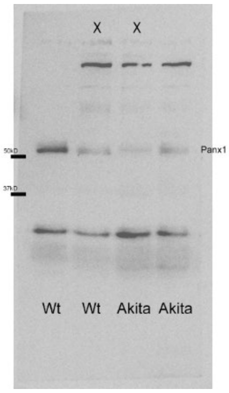

β-actin

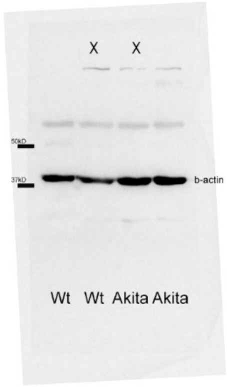

P2X7R

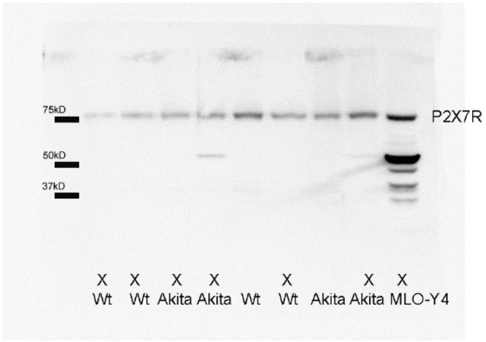

β-actin

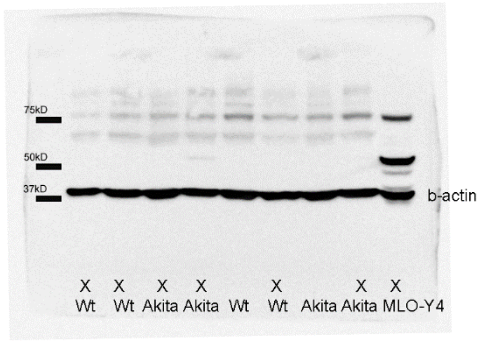

10W Panx1, P2X7R, and the corresponding β-actin loading controls

Panx1

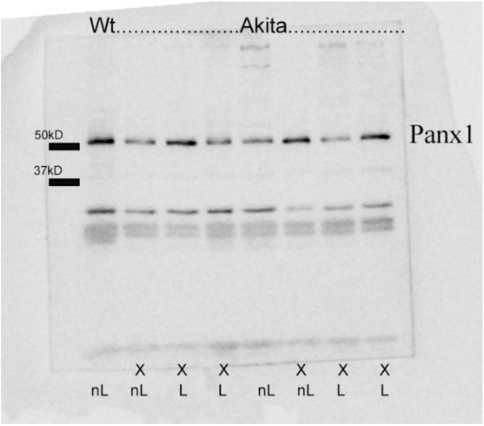

β-actin

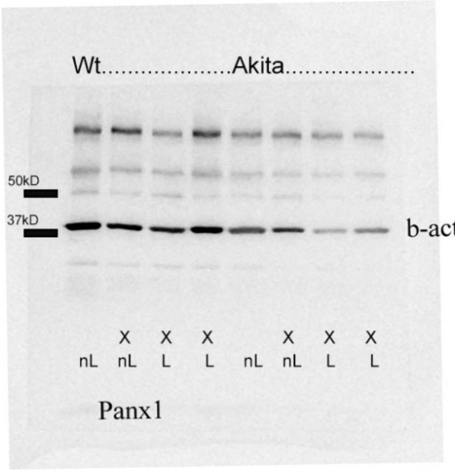

P2X7R

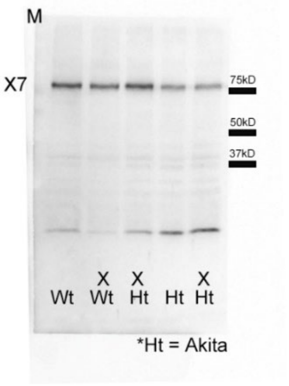

β-actin

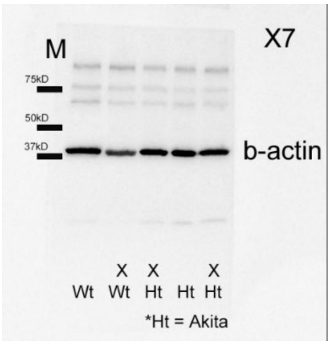

12W Panx1, P2X7R, and the corresponding β-actin loading controls

Panx1

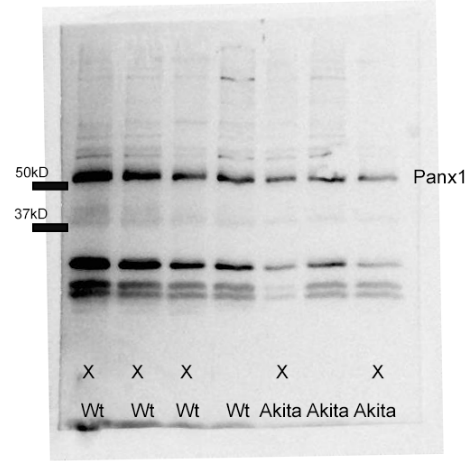

β-actin

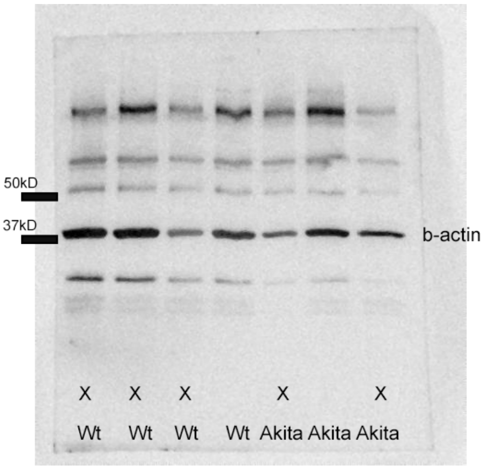

P2X7R

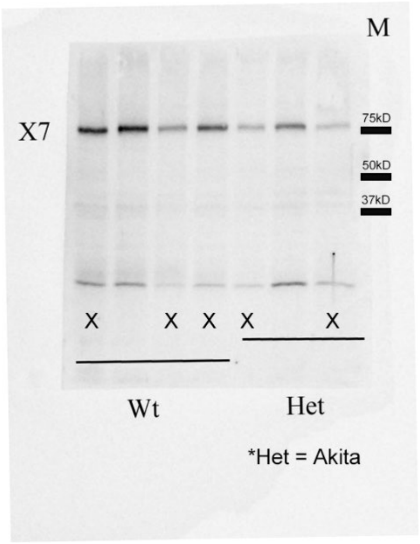

β-actin

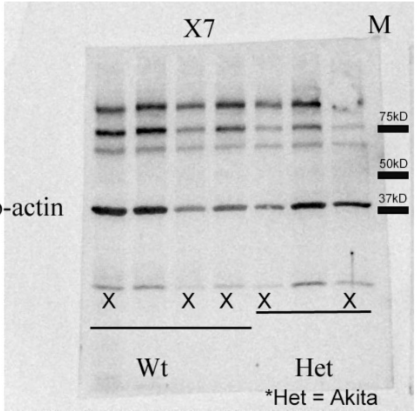

Figure 4

X: Denotes lanes not used as representative blots in the figure
nL: non-loaded samples, L: loaded samples

1 week loading: Panx1, P2X7R, and the corresponding β-actin loading controls

Panx1

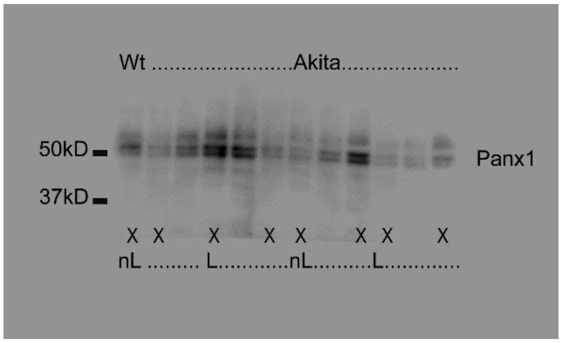

β-actin

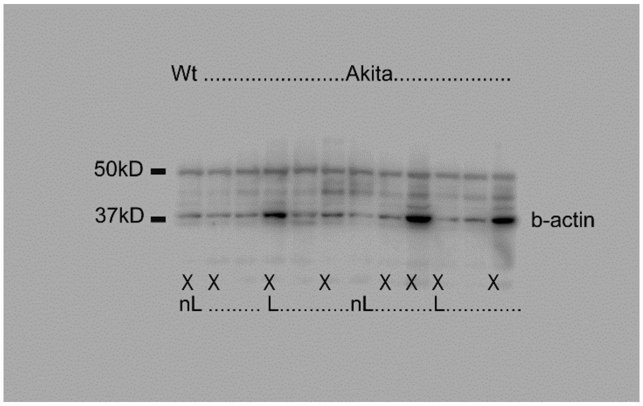

P2X7R

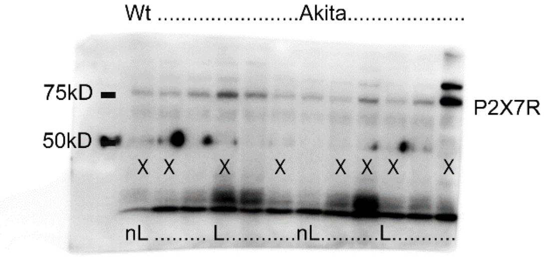

β-actin

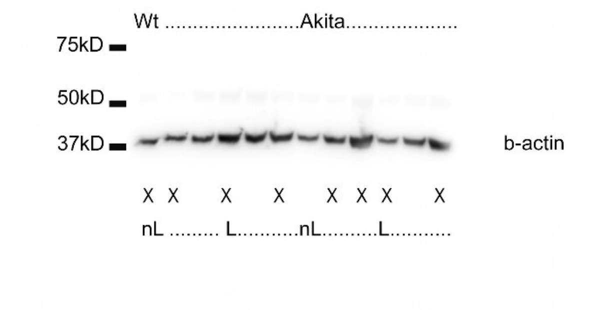

2 weeks loading: Panx1, P2X7R, and the corresponding β-actin loading controls

Panx1

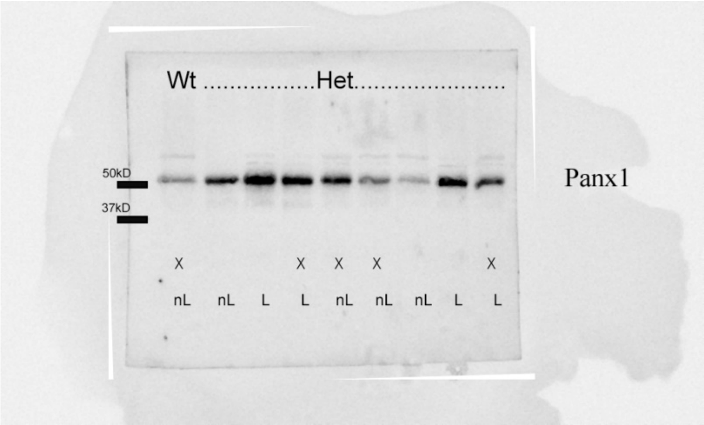

β-actin

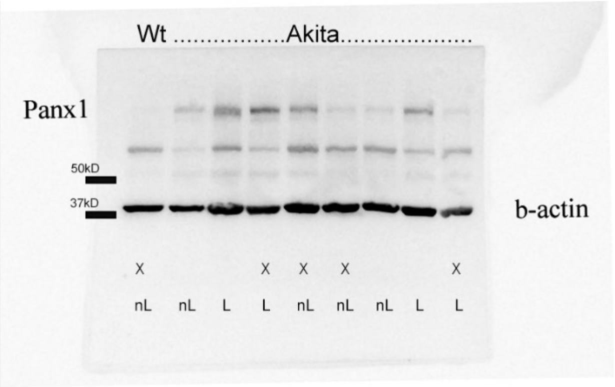

P2X7R

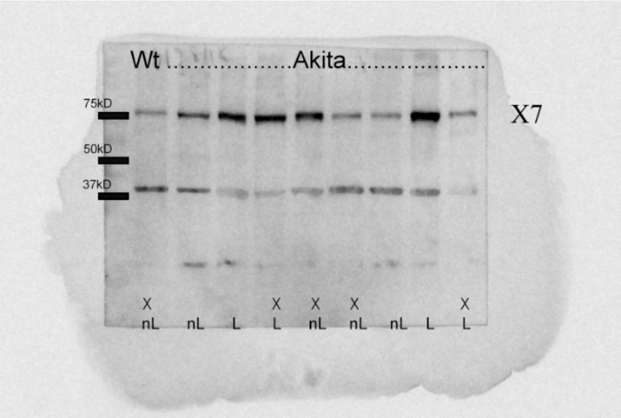

β-actin

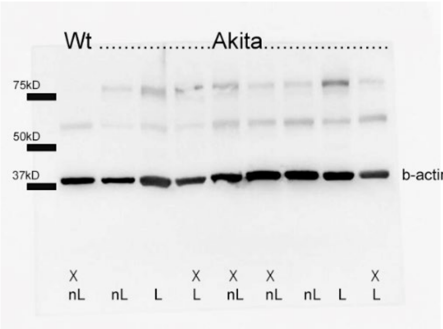

4 weeks loading: Panx1, P2X7R, and the corresponding β-actin loading controls

Panx1

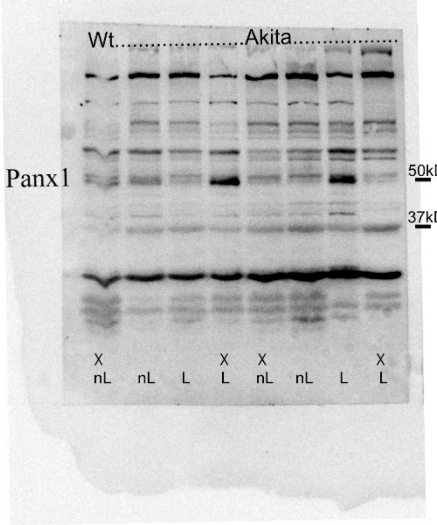

β-actin

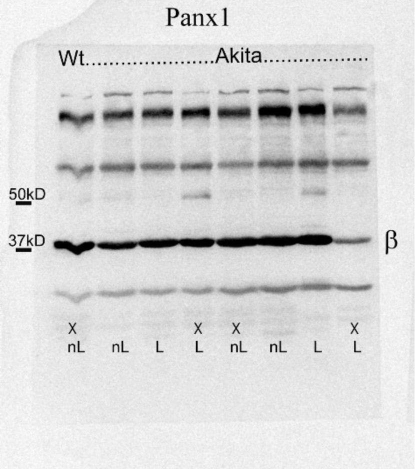

P2X7R

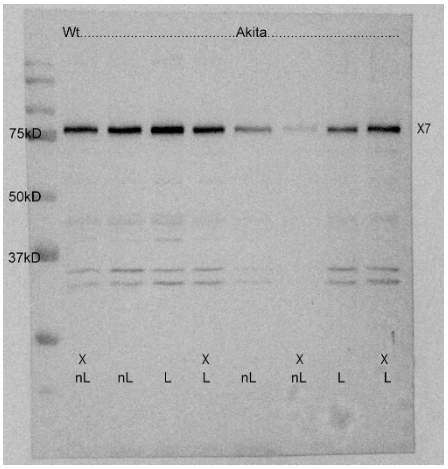

β-actin

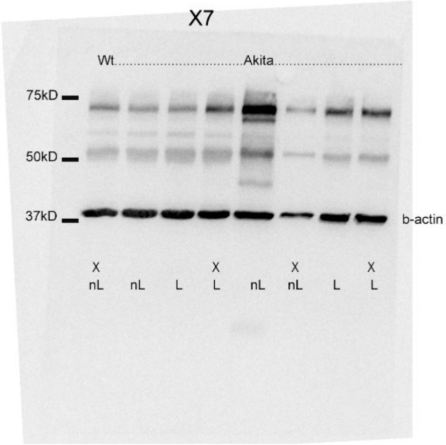

Supplement: Supplementary file 1 [file DataSheet1.pdf]
